# Acknowledgement to Reviewers of *Molecules* in 2017

**DOI:** 10.3390/molecules23010138

**Published:** 2018-01-10

**Authors:** Molecules Editorial Office

**Affiliations:** MDPI AG, St. Alban-Anlage 66, 4052 Basel, Switzerland; molecules@mdpi.com

Peer review is an essential part in the publication process, ensuring that *Molecules* maintains high quality standards for its published papers. In 2017, a total of 2242 papers were published in the journal. Thanks to the cooperation of our reviewers, the median time to first decision was 15 days and the median time to publication was 36 days. The editors would like to express their sincere gratitude to the following reviewers for their time and dedication in 2017:
Aaron, Jean E.Løvdal, TrondAaronson, Philip I.Loveridge, JoelAbad-Fuentes, AntonioLozano-Juste, JorgeAbarbri, MohamedLu, Chi-YuAbashev, G. G.Lu, DiannanAbbamondi, Gennaro RobertoLu, GuifenAbd-El-Aziz, AlaaLu, Hsu-FengAbdelaziz, MohamedLu, JianAbe, HiroshiLu, Jyh-FengAbe, KentaroLu, Mei-ChinAbourashed, EhabLu, RongAbraham, E. M.Lu, ShenzhouAbraham, NaderLu, SizhaoABUGRI, DANIELLu, XiaoAbul-Haija, YousefLucas, GuilhermeAbu-Reidah, Ibrahim M.Lucena, RafaelAceña, Jose LuisLudwiczuk, AgnieszkaAcero, N.Lugo-Cervantes, EugeniaAchelle, SylvainLugo-Cervantes, Eugenia Del CarmenAcuña-castroviejo, DarioLühder, FredAdam, VojtechLuk, FrederickAdamczyk-Woźniak, AgnieszkaŁukomksa-Szymańska, MonikaAdegoke, Anthony AyodejiLuo, Huai-RongAdhikari, UpendraLuo, LuGuangAdhikary, AmitavaLuongo, LivioAdunyah, Samuel E.Lupiáñez, José AntonioAfaq, FarrukhLupien, AndréanneAfonin, KirillLupták, AndrejAfonso, Clélia N.Luque, F. JavierAfzal, MohammedLushington, GerryAgamennone, MariangelaLustri, WiltonAgarwal, RajeshLutza, Cathleen M.Agarwal, VinayakLuyt, LeonardAgbaba, DanicaLuzinia, OlgaAgirre, JonLymperopoulos, AnastasiosAgostino, MarkLyutakov, OleksiyAgrahari, VivekM. S. Silva, ArturAgrawal, PrashansaM.Pharm., Yagna PRAgut, MontserratMa, CongAherne, CMMa, MichelleAhmad, NihalMa, WeiAhmad, ZeeshanMa, XingmaoAhmed, AlauddinMa, XingzheAhmed, HafnaMacabeo, Allan P.Ahn, Tae SeokMaccallini, CristinaAiba, YuichiroMacchi, PieroAiello, FrancescaMacernis, MindaugasAitken, AlanMach, MojmirAitor, Nogalez-GonzalezMachado, PabloAkamatsu, MikiMacheroux, PeterAkhavan, OmidMachetti, FabrizioAkhtari, MassoudMaciejewska, DorotaAkine, ShigehisaMackay, SimonAkk, GustavMaczka, MirosławAlabugin, IgorMadala, Ntakadzeni EAlaimo, SalvatoreMaddau, LuciaAlam, Mohammad AbrarMadesis, PanagiotisAlam, Mohammad ParvezMadrid, AlejandroAlbada, BaukeMaeda, ChihiroAlbericio, FernandoMaeda, HayatoAlbini, AngeloMaeda, KazuyaAlbiniak, PhilipMaehre, Hanne K.Alcolea Palafox, MauricioMaestri, MatteoAl-Dujaili, EmadMaestro, MiguelAlegre, CinthiaMagalhães, Júlia M.C.S.Alevizopoulos, KonstantinosMagalhães, Washington Luiz EstevesAlexiou, ChristophMagata, YasuhiroAlfano, Orlando MarioMaGee, DavidAl-Fatimi, MohamedMaggi, FilippoAlferink, JudithMaggioni, DanielaAl-Horani, RamiMagid, Abdulmagid AlabdulAli, Hamed I.Magiorkinis, EmmanouilAli, Md AzaharMagnaldo, ThierryAliverti, AlessandroMagnier, EmmanuelAllais, FlorentMagoulas, GeorgeAller, PatricioMagoulas, George E.Allwood, Daniel M.Magriotis, Plato A.Almajano, María PilarMaher, PamelaAlmeida, HugoMahimainathan, LeninAlmquist, CatherineMahmudov, KamranAl-Mutairi, MahaMahuteau-Betzer, FlorenceAltarawneh, MohammednoorMaia Santos, Roseane MariaAltemimi, AmmarMAICAN, EdmondAltieri, FabioMaier, VítězslavAlvarenga, ElsonMaione, FrancescoÁlvarez, María S.Majinda, Runner R.T.Alvarez-Dominguez, CarmenMajkowska-Skrobek, GrażynaAlves, Maria JoséMajtan, JurajAmaki, WakanoriMajumdar, SusrutaAmarowicz, RyszardMäkelä, Jyrki M.Amiche, MohamedMakino, YoshioAmin, Mohammed NMalacarne, GiuliaAmiral, JeanMalde, AlpeshAmmendola, RosarioMaldini, MariateresaAmmu, PrhashannaMallavia, R.Amorati, RiccardoMallipeddi, Prema LathaAn, JiaMalmström, LarsAnas, Andrea RoxanneMalterud, KarlAnderson, Anne J.Malucelli, GiulioAndersson, UlfMamane, VictorAndjelkovic, Anuska V.Mamat, ConstantinAndjelkovic, MirjanaMammi, StefanoAndolfi, AnnaManabe, KeiAndrade, ArturoManavalan, BalachandranAndrade, Paula B.Mancheño, JoséAndrea, PenoniMancini, InesAndreoli, EnricoMancini, RockAndreoli, RobertaMancuso, RaffaellaAndric, FilipMandal, BivashAndrieux, FabriceMandalari, GiuseppinaAnfossi, LauraManfredi, Kirk P.Angeletti, MauroManfredi, NorbertoAngelini, GuidoManfredini, StefanoAngelino, DonatoMang, Thomas S.Angelova, Violina T.Mangalagiu, IonelAnglister, JacobMangayil, RahulAnna Maria, FerrariMangunuru, PrasadAnna, Pachuta-StecManjunath, ManuboluAnsquer, Jean ClaudeMann, FrancisAntal, Diana SimonaMannell, HannaAnthony, BenMano, YujiAnton, SteveManos, ManolisAntonio, Rodríguez-ForteaMansoorabadi, Steven O.Anzai, JunichiMante, Francis K.Anzai, Jun-ichiMantellini, FabioAoki, YoshitsuguMantovani, GiuseppeApfel, Ulf-PeterManzano, Blanca R.Appell, MichaelMao, ChuanbinAppendino, GiovanniMao, JiaAprea, EugenioMapelli, ValeriaArancibia, RodrigoMarangon, MatteoArango, Rachel A.Marasco, DanielaAraniciu, CătălinMarchand, PascalArchibald, SteveMarchetti, NicolaArgia, KatsuhikoMarchini, CristinaArgyri, Anthoula AMarco-Contelles, JoséArgyropoulou, AikateriniMarcotullio, CarlaArmanini, DecioMarczak, LukaszArmeli Minicante, SimonaMargetic, DavorArmitage, BruceMargina, DenisaArnason, JohnMarhuenda, JavierArndt, Patrick G.Márialigeti, KárolyAros, DaniloMaric, MilanArsenian-Henriksson, MarieMarichev, KostiantynArtés-Hernández, FranciscoMarin, DanielaArvapally, Ravi KumarMarin, Jose J. G.Asahara, HaruyasuMarini, FrancescaAsai, AkiraMarino, AttilioAsai, DaisukeMarino, JosephAsakawa, YoshinoriMarkiewicz, Wojciech T.Asakura, TetsuoMarkiv, AnatoliyAsensio, Juan LuisMarkopoulou, OlgaAsfin, Ruslan E.Marques, M. Manuel B.Ashida, HitoshiMarra, AlbertoAsia, Haji AkberMarrocchi, AssuntaAslam, MuhammadMarrucho, Isabel M.Aspatwar, AshokMartell, MariaAssis, Odilio Benedito GarridoMartelli, Pier LuigiÅstrand, AlexanderMartí, SergioAsuero, Agustín G.Martin Lawson, AtaAttard, ThomasMartín, Antonio E.Auberon, FlorenceMartin, FinianAudette, Gerald FMartin, JessicaAureliano, ManuelMartin, PatriciaAury-Landas, JulietteMartín-Aragón, SagrarioAU-YEUNG, Ho YuMartinelli, PaolaAvarvari, NarcisMartínez, AlbertoAvato, PinarosaMartinez, AnaAverbeck, DietrichMartínez, AntonioAverina, E. B.Martínez, Jaime RubioAvinoam, OriMartinez, Jose LAvram, Anca M.Martinez, ManuelAyala, Alejandro PedroMartínez-Campa, CarlosAyala-Zavala, J. FernandoMartínez-Espinosa, Rosa MaríaAyotte, ChristianeMartin-Gil, V.Azab, AbedMartins, LuísaAzimi, ImanMartins, Maria RosarioAzqueta, AmayaMartins, NatáliaAzuma, KazuoMartí-Rujas, JavierBaba, YuhMaruyama, KeiBabaev, EugeneMarverti, GaetanoBack, K.Mary, DidierBacso, ZsoltMarzaro, GiovanniBae, Soo KyungMarzocco, StefaniaBaek, Nam-InMarzouk, Magda I.Baerga-Ortiz, AbelMascal, MarkBaeza, AlejandroMascetti, JoëlleBaggiani, ClaudioMasek, AnnaBaginski, MaciejMaserti, Bianca ElenaBaglio, VincenzoMashima, RyuichiBai, RenrenMasino, FrancescaBai, ShiqiangMasłyk, MaciejBai, ShuhuaMason, William S.Bai, YanfenMassa, AntonioBajpai, RichaMasson, PatrickBak, IstvanMassoud, Tarik F.Baker, C. JacynMastrangelo, Anna M.Baker, Murray V.Masu, HyumaBakunina, IrinaMata, IgnasiBal, WojciechMatczak-Jon, EwaBalasubramaniam, MeenakshisundaramMateo, CesarBalch, AlanMathesius, UlrikeBaldoví, Jose J.Mathews, L. DalilaBalduini, WalterMathieu, DBaleja, JimMatiadis, DimitrisBali, ShilpaMatilla, Miguel A.Bálint, ErikaMatkowski, AdamBall, K. AureliaMatsoukas, Minos-TimotheosBalova, IrinaMatsuda, HisashiBaltriukiene, DaivaMatsuda, YudaiBaluska, FrantisekMatsugo, SeiichiBamdad, CynthiaMatsui, DaisukeBanach, MarcinMatsukawa, SatoruBandi, VenuMatsumoto, KozoBandyopadhyay, SulalitMatsumoto, ShojiBanerjee, AditiMatsumoto, YasuhikoBaniahmad, AriaMatsumura, KiyoshiBankova, VassyaMatsumura, ShuichiBannach, OliverMatsuo, HiroshiBanskota, Arjun H.Matsuo, TakashiBanti, Christina N.Matsuo, YosukeBao, GuanhuMatsuura, BunzoBao, YongpingMatsuura, HiroshiBapputty, ReenaMattei, FabrizioBarakat, KhaledMatthews, SusanBaranski, RafalMattoo, AutarBarba, FranciscoMatwijczuk, ArkadiuszBarbaud, ChristelMaurer-Jones, MelissaBarczyński, PiotrMavila, SudheendranBardají, EduardMavromoustakos, ThomasBarendt, TimothyMaxwell, AnthonyBarenys, MartaMazerska, ZofiaBarker, DavidMazzaglia, AntoninoBarla, ThirupathiMazzei, PierluigiBarlow, Peter G.Mazzone, GloriaBarnathan, GillesMazzoni, LucaBarnett, MatthewMbosso, Emmanuel Jean TeinkelaBarraja, PaolaMcAdam, Scott A. M.Barrajón-Catalán, EnriqueMcardle, PatrickBarraud, NicolasMcCallum, Jason L.Barroso-Bogeat, AdriánMcCarthy, James B.Barta, CsengeleMcCarthy, Thomas J.Bartelt, AlexanderMcClain, William H.Bartelt, NormanMcConnell, Erin M.Bartocci, PietroMcDaneld, Tara G.Bartolini, ManuelaMcDonald, KarenBartolini, PaoloMcdougal, Owen M.Bartolomé, BegoñaMcDougall, GordonBartosz, GrzegorzMcEvoy, JamesBartunek, VilemMcFeeters, Robert L.Basco, Leonardo KMcGarrigle, Eoghan M.Basiak, EwelinaMcGillivray, ShaunaBasini, GiuseppinaMcGlinchey, Michael J.Bassin, PaulMcGonigal, Paul R.Batista, IrineuMcHenry, PeterBattino, MaurizioMcKeague, MaureenBattistelli, CeciliaMcLaughlin, EmilyBauer, EikeMcLaughlin, MarkBaum, ChristelMcMillin, Kenneth W.Bavaresco, LuigiMcMillin, MatthewBavaro, TeodoraMcRae, Jacqui M.Beale, MichaelMeade, ThomasBeato, PabloMedici, SerenellaBębenek, EwaMedina, MilagrosBeberok, ArturMedio-Simón, MercedesBecker, Donald F.Medvedovici, AndreiBednarz, SzczepanMehbub, Mohammad FerdousBedse, GauravMehrotra, ShikharBei, RobertoMehta, Akul Y.Bejarano, IgnacioMei, BastianBekbolet, MirayMeier, SebastianBeld, JorisMeijide, FranciscoBelfield, Kevin D.Mejías, MarcBelford, RobertMeldolesi, JacopoBellés, José MaríaMelguizo-Guijarro, ManuelBellich, BarbaraMeli, RosariaBello, Nicholas T.Melini, ValentinaBellou, StamatiaMella, JaimeBełtowski, JerzyMellander, LisaBencsik, TimeaMelo, AndréBendas, GerdMelo, Manuel N.Bendicho, CarlosMelone, LucioBen-Dov, Iddo Z.Melzig, Matthias F.Benedec, DanielaMendel, MartaBenetatos, PanayotisMendes, Adriano AguiarBenhida, RachidMendes, Ana C.Benito, Juan M.Méndez Bolaina, EnriqueBenoist, EricMéndez-Sánchez, NahumBenoit, David M.Menendez, EstherBeppu, FumiakiMenet, Marie ClaudeBerhow, MarkMeng, XiaomingBerlanga, Ángel MéridaMenghini, LuigiBerlin, K. DarrellMenna, MarialuisaBerlowska, JoannaMerah, OthmaneBermudez, MarcelMercader, JosepBernini, RobertaMerigo, FlaviaBerreau, Lisa M.Merino, GabrielBerrocal-Lobo, MartaMerlini, LucioBerski, WiktorMeschwitz, SusanBertaccini, Edward J.Messina, Concetta MariaBertani, RobertaMessyasz, BeataBerti, FedericoMetelmann, Hans-RobertBertran, JoanMetzinger, LaurentBerzal-Herranz, AlfredoMeulenberg, ElineBesalú, EmiliMewis, IngaBessa, LucindaMeyer, Anne S.Besson, ThierryMeyer, JörgBethanis, KostasMezei, GellertBetoret, EsterMézes, MiklósBettini, SimonaMezeyova, IvanaBevilacqua, ArturoMichaelis, David J.Beyeh, NgongMichalcova, LenkaBeyeh, Ngong KodiahMichalska, AnnaBezirtzoglou, EugeniaMichel, PiotrBharadwaj, VivekMicheletti, GabrieleBhat, SaleemMidgley, AdamBhattacharya, DebswapnaMiecznikowski, K.Bhattacharya, SoumyaroopMiele, Adriana E.Bhattacharyya, GargiMiguel, GraçaBhaumik, AnusarkaMíguez, Jesús M.Bhullar, Rajinder P.Miki, KojiBhusal, Siddhi JeewanMiki, YasuhiroBhutoria, SavitaMiklossy, GabriellaBiagini, GiuseppeMikołajczyk-Bator, KatarzynaBiałek, MarzenaMikstacka, RenataBianco, ArmandodorianoMikulski, DamianBianco, I.D.Milano, FrancescoBiasutto, LuciaMilej, DanielBidel, LucMilelli, AndreaBiedermann, DavidMiller, JohnBielenica, AnnaMillet, OscarBiernasiuk, AnnaMillot, MarionBiesaga, MagdalenaMimaki, YoshihiroBijak, MichałMimura, TetsuroBikiaris, DimitriosMinami, MasabumiBilusic, TeaMinami, YasunoriBirch, JohnMinato, Ken-ichiroBirkemeyer, ClaudiaMinbiole, Kevin P. C.Birney, David M.Mindt, Thomas L.Bischof, JoachimMinervini, GiovanniBishayee, AnupanMinjares-Fuentes, Jose RafaelBishop, Anthony C.Minkiewicz, PiotrBishop, RogerMiranda, CristobalBishop-Hurley, SharonMiranda, Jose M.Bisignano, GiuseppeMisaki, TomonoriBišová, KateřinaMiseki, YugoBisson, JonathanMishra, NigamBisti, SilviaMishra, Rupesh K.Bizukojc, MarcinMislin, Gaëtan L.A.Bizzarri, Anna RitaMitani, TakakazuBizzarri, MarianoMitchell, Scott G.Bjorninen, MiinaMitsudo, KoichiBjornstedt, MikaelMittelberger, FlorianBlais, AnneMiura, TsuyoshiBlasiak, JanuszMiyabe, HidetoBłaszczyk, JarosławMiyamoto, KojiBłażewska, Katarzyna M.Miyawaki, OsatoBleackley, Mark R.Miyazato, HironariBoard, MaryMizuno, Cassia SBoddy, Christopher N.Mizuno, MasashiBoehr, David DMizutani, OsamuBoga, CarlaMladěnka, PřemyslBogel-Łukasik, RafałMlcek, JiriBogialli, SaraMłochowski, JacekBogliotti, NicolasMnif, WissemBøgwald, JarlMocan, AndreiBöhm, VolkerMochona, BereketBohn, TorstenModec, BarbaraBojić, MirzaModica, MariaBojková, BiankaModos, DezsöBolibok-Brągoszewska, HannaMoen, Aina E.F.Bolivar, Juan M.Mogi, MasakiBondon, ArnaudMohankumar, KumaravelBonetta, SilviaMohr, Justin T.Bongarzone, SalvatoreMoineau-Chane-Ching, Kathleen I.Bongiorno, DavidMok, Daniel Kam-WahBonifácio, VascoMolina, MariaBonikowski, RadosławMolinari, FrancescoBonneau, NatachaMöller, HeikoBonnet, VeroniqueMollica, AdrianoBonny, OlivierMoloney, MarkBonvicini, FrancescaMoncayo, RoyBora-Singhal, NamrataMonchau, FrancineBorawska-Dziadkiewicz, JustynaMonchaud, DavidBorbas, K. EszterMonclus, MichelBorbone, NicolaMondal, AnupomBoros, LaszloMondello, LuigiBorota, AnaMonflier, EricBort, Juan M. AndrésMonfort, Xavier SolansBoryczka, StanisławMonika, Waksmundzka-HajnosBosca, FranciscoMoniuszko-Szajwaj, BarbaraBosch, PaulaMontana, Angel M.Boschin, GiovannaMontenarh, MatthiasBose, DebojitMontenegro, LuciaBose, SayantanMonti, Daria MariaBose, UtpalMonti, Maria ChiaraBosnjak, BerislavMoog, ChristianeBosso, LucianoMoon, Byeong CheolBotana, Ana M.MOON, ILSOOBotta, BrunoMoraes, Valéria R SBotta, MaurizioMorais, VictorBottle, SteveMorales-Narváez, EdenBouquillon, SandrineMoreira Borges, RicardoBourgeois, DamienMoreira, Irina S.Boutin, JeanMoreira, JoséBowden, BruceMorelli, MicaelaBowden, GregoryMorel-Rouhier, MélanieBoyer, François-DidierMoremen, KelleyBoylan, FabioMoreno, AndrésBožović, MijatMoreno, Antonio D.Braakhuis, AndreaMoreno, J.J.Brachet, EtienneMoreno, Juan CarlosBradshaw, PatrickMoreno, SilviaBraig, SimoneMorganti, PierfrancescoBranca, FerdinandoMori, MattiaBranca, MathieuMorigi, RitaBranchini, AlessioMorikawa, ToshioBrand, JacoMorimura, ShigeruBravo López, Susana BMorita, KyojiBrazzolotto, XavierMorkunas, IwonaBreaker, RonaldMorley, KristaBrebu, MihaiMoro, StefanoBrecker, LotharMoroshkin, PeterBreinbauer, RolfMorrill, Louis C.Bren, UrbanMorris, GordonBreschi, AlessandraMorris, James C.Brestic, MarianMorrison, DouglasBriedis, VitalisMorsch, MarcoBrinchmann, Monica F.Mortha, GérardBrindisi, MargheritaMosawy, SaphaBrinker, C. JeffreyMoschos, Sterghios A.Broberg, AndersMoschou, Panagiotis N.Brogi, SimoneMota, ManuelBrouwer, A.M.Moudgil, KamalBru, RoqueMourdikoudis, StefanosBrück, ThomasMourtas, SpyrosBruckner, ChristianMoyano, AlbertBrudzynski, KatrinaMoynihan, Humphrey A.Brullo, ChiaraMoynihan, PatrickBrumm, Phillip J.Mshvildadze, VakhtangBrun, RetoMučaji, PavelBrunauer, GeorgMuccilli, VeraBrunetti, CeciliaMuceniece, RutaBrycki, BogumiłMucha, ArturBrzozowski, TomaszMudit, MuditBuchet, ReneMuggia, LuciaBuchowicz, WłodzimierzMugo, SamuelBuckley, M.Muhle-Goll, ClaudiaBudillon, AlfredoMühlenhoff, UlrichBudryn, GrazynaMukai, HidehitoBudzak, SimonMukherjee, SubhenduBudzisz, ElzbietaMulas, MaurizioBueken, BartMuller, C.J.F.Bufo, Sabino A.Muller, Christian D.Bugarin, AlejandroMüller, ChristophBugg, Timothy D.H.Müller, KarstenBuksa, KrzysztofMuller, MarcBulgariu, LauraMüller, Thomas J. J.Bullon, PedroMüller, WernerBulow, LeifMüller, Werner E.G.Bun NG, TziMulloy, BarbaraBunaciu, Rodica PMun, JiyoungBunce, Richard A.Munang’andu, Hetron MweembaBurcul, FrankoMunteanu, Bogdanel SilvestruBurkitt, MichaelMura, UmbertoBurley, Jonathan C.Murai, ToshiakiBurow, MeikeMurali, D.Burzuri, EnriqueMurata, ToshihiroBusinelli, DanielaMurelli, Ryan P.Busqué, FélixMuresan, VladBusquets, Maria AntòniaMurkovic, MichaelButtachon, SuradetMurotomi, KazutoshiButterweck, VeronikaMurphree, S. ShaunButterworth, Peter J.Murphy, AndrewBuxton, DenisMurray, JaneByrne, JohnMurray-Stewart, TracyBystricka, JuditaMusch, MarkBystrowska, BeataMusiol, RobertCabral, CéliaMusso, LoanaCabral, HoracioMustafa, S. JamalCabrales, LuisMutić, JelenaCabrele, ChiaraMutlu, HaticeCabrita, Maria JoãoMyllykallio, HannuCadierno, VictorioMysliwiec, JaroslawCádiz Gurrea, María De La LuzMysliwiec, TamiCaelles Franch, CarmenN. Scott, LitofskyCaffarel, María MuñozNaal, RoseCaffrey, PatrickNadolna, JoannaCafiero, MauricioNagai, KatshitoCAGLIERO, Cecilia LuciaNagai, YoshinoriCaiazzo, ElisabettaNaganawa, YukiCaira, Mino R.Nagaraj, Vinay J.Cairo, GaetanoNagarajan, SureshbabuCairrão, ElisaNagasawa, KazuoCalabrese, FrancescaNagata, EiichiroCalcabrini, CinziaNagata, ToshiCalcutt, MichaelNagatsu, AkitoCalderone, VincenzoNagatsugi, FumiCallahan, Laura SmithNagy, PeterCallebaut, IsabelleNair, Ashwin M.Callery, Patrick S.Nair, VimalCalvete, Juan J.Najlah, MohammadCalvete, MarioNakagawa-Goto, KyokoCalvo, FlorentNakajima, MasatoshiCâmara, JoséNakamura, TsukasaCamblor, Miguel A.Nakano, HideyukiCamele, IppolitoNakashima, SouichiCamerel, FranckNakata, NorioCametti, C.Nakayama, TsutomuCampagne, Jean-MarcNakayama, YujiCampana, RaffaellaNakielski, PawelCampanari, Maria-letiziaNalewajko-Sieliwoniuk, EdytaCampbell, ChristopherNaliwajko, SylwiaCampbell, Grant R.Namiesnik, JacekCampbell, LeeNandakumar, Kutty SelvaCampillo, Nuria E.Nandha Premnath, PadmavathyCampo, GiuseppeNandwana, VikasCampos Rosa, JoaquínNaponelli, ValeriaCanal, José MªNarayanan, AnandCañas, Rafael A.Narayanan, GaneshCandeias, Nuno R.Nardi, MonicaCandela, HéctorNarsimhan, GanesanCandiani, SimonaNarváez, ManuelCanela, RamonNasri, RimCaneschi, AndreaNass, NorbertCanini, AntonellaNastasa, CristinaCannilla, CatiaNatarajan, ArutselvanCao, Qing-RiNatile, GiovanniCao, RenzhiNaumovski, NenadCao, ShuwenNavarra, MicheleCao, TongyuNavío, Jose AntonioCapasso, RaffaeleNavrot, NicolasCappellin, LucaNawirska-Olszańska, AgnieszkaCapriati, VitoNawrocka, AgnieszkaCarabineiro, Sonia A. C.Nawrot, BarbaraCaramujo, Maria JoséNazaruk, JolantaCardinali, Daniel P.Nazzaro, FilomenaCardona, FrancescaNedachi, TakuCardoso, SusanaNedialkov, Paraskev TodorovCardoso, Susana M.Nee, Matthew J.Caretti, AnnaNeely, Brian J.Carillo, PetroniaNeergheen-Bhujun, VidushiCarlsson, JensNegishi, NobuakiCarmagnola, IreneNeilson, AndrewCarocho, MárcioNelson, Fred R.T.Caroli, Anna MariaNemoto, KiyomitsuCarosio, FedericoNemykin, VictorCarpene, ChristianNeng, Nuno R.Carradori, SimoneNepal, MadhavCarrasco, HéctorNepveu, FrançoiseCarrasco, SergioNerva, LucaCarta, FabrizioNesmelov, Yuri E.Caruso, GerardoNesmelova, IrinaCarvajal, MicaelaNeumann, TerrenceCasado-Coterillo, ClaraNeunert, GrazynaCasalino, GabriellaNeureiter, DanielCasassa, L. FedericoNevárez-Moorillón, Guadalupe VirginiaCasassa, Luis FedericoNg, AlphonsusCascioferro, StellaNG, Tzi BunCasellini, CarolinaNgai, Ming-YuCassidy, JohnNguyen, Duc ThanhCastanho, MiguelNiamké, SébastienCastell, AlinaNicoletti, FerdinandoCastellano, FlaviaNie, MengyanCastñeiras, AlfonsoNiehaus, TomCastrén, Maija L.Nielsen, Line HagnerCastro Ruiz, LauraNielsen, Vance G.Castro, MariaNieminen, KaarloCastro, Mariana S.Nieto, PérezCattaneo, FabioNiimi, JunCatto, MarcoNikolaidis, NikolaosCavaleiro, JoséNikolakakis, IoannisCavalli, RobertaNikolic, DejanĆavar Zeljković, SanjaNikolic-Paterson, DavidCejka, JiriNikonowicz, Edward P.Celeiro, MaríaNilsson, R. HenrikCellamare, SaverioNishimoto, YoshioCen, ShanNishiwaki, HisashiČėnas, NarimantasNishizawa, SeiichiCenci, ElioNisnevitch, MarinaCentonze, DiegoNitulescu, George MihaiCeranowicz, PiotrNogueira Perez, Juan JoseCerella, ClaudiaNolte-t Hoen, Esther N. M.Cerra, BrunoNonami, AtsushiCeruti, StefaniaNonappa, NonappaCervantes, JairNonnemacher, Michael R.César, VincentNorman, TrevorChackerian, BryceNotestein, JustinChacón, PabloNotni, JohannesChae, HeeyoungNovo, EricaChaerle, LauryNowak, SaschaChaimbault, PatrickNoyes, Richard D.Chakraborty, IndranilNtalli, NikolettaChan, Ben C. L.Ntalli, Nikoletta G.Chan, Yu-YiNugen, Sam R.Chandaka, Giri KumarNunes, CláudiaChang, Chia MingNunzio, Mattia DiChang, Ching-yaoNurieva, EvgeniyaChang, Chuang-RungO’Shea, KevinChang, Fang-RongObata, YasukoChang, Hsueh-WeiOćwieja, MagdalenaChang, JungshanO’Doherty, GeorgeChang, Shang-TzenOehlers, StefanChang, Wei-JenOga, Enoche F.Chang, Wen-WeiOgawa, AkiyaChang, Yan ShiunOgawa, NorikoChao, Chih-HuaOgawa, ShojiroChao, Louis KuopingOgihara, JunChao, Pi-YuOgino, ShujiChappell, JosephOgnyanov, ManolCharles, Prof. Dr. EdwinOgra, YasumitsuCharlton, ClivelOgungbe, IfedayoChatterjee, JhinukOh, DongchanChavez, FermanOh, Kyung TaekChazin, Walter J.Oh, KyungsooChe, Chun-TaoOh, Sei-RyangChe, LixiaoOh, Won KeunChemat, FaridOh, Yoon SinChen, BanglinO’Hagan, DavidChen, Bing-HungOhashi, NaroChen, ChaoOhkanda, JunkoChen, ChiachungOhlson, MikaelChen, ChunchengOhnishi, KohtaChen, Chung-YiOhsaki, AyumiChen, Chun-JungOhta, ShinjiChen, FushengOikawa, AkiraChen, GangOishi, TohruChen, Gen-HungOjala, Johanna O.Chen, GuoxunOkada, TaketoChen, HanqingOkamoto, MasamiChen, HaotongOkamoto, ShigefumiChen, HouyangOkazawa, AtsushiChen, Hsin-ChunO’Keefe, Barry R.Chen, HuaiqiongOkesola, BabatundeChen, HuixiongOKTE, A. NerenChen, Jhy-DerOkunade, Adewole L.Chen, Jiann-ChuOláh, AttilaChen, Jih-JungOlaru, MihaelaChen, JihuaOlech, MartaChen, JinquanOliva, LeoneChen, Kew-YuOliva, RominaChen, Kuo-YuOliveira Rocha, Hugo AlexandreChen, Lih-GeengOliveira, Cláudia SirleneChen, LuluOliveira, Hugo M.Chen, Lung-ChienOliveira, José M.Chen, Miaw-LingOliveira, LauraChen, MohanOliveira, Paula Alexandra Martins DeChen, Nai-HongOliveira, RuiChen, QiOliver, Allen G.Chen, Qiu-YunOliverio, ManuelaChen, ShangwuOliviero, GiorgiaChen, WanzhiOlkkonen, Vesa M.Chen, XiaolanOller, IsabelChen, XiaozhuoOllila, SamuliChen, YiliangOllis, DavidChen, YinghuiOlson, KennethChen, Yun-WenOlszewska, Monika AChen, ZhihangOmbra, Maria NeveChen, ZhongOmosa, Leonidah K.Cheng, Chih-ChiaOms, OlivierCheng, ChongOms-Oliu, GemmaCheng, HuanyuOnduka, ToshimitsuCheng, Juei-TangO’Neil, GregoryCheng, KaiOng, Eng ShiCheng, Kuan-ChenOniga, IlioaraCheng, Qing-QingOniszczuk, AnnaCheng, Sen-SungOnnis, ValentinaCheng, WaiwaiOno, TeruhishaCheng, XingguoOnuki, AkiraCheng, XinlaiOnyenwoke, Rob UCheng, XuOpel, OliverCheng, YinjiaOri, OttorinoCheriyath, VenugopalanOrian, LauraCHERKAOUI MALKI, MustaphaOrtega, NatividadChern, Ming-KaiOrtelli, SimonaCherniack, Evan PaulOrtiz, Gregorio FChetwynd, AndrewOrts, JulienChevrot, RomainOrtuno, ManuelCheynier, VeroniqueOsadolor, Osagie A.Chiadò, AlessandroOsanai, TomohiroChiang, Anthony S.T.Oschatz, MartinChiang, Ming-HsiOsolodkin, DmitryChibalin, Alexander VOspina Millan, ClaudiaCHIEN, HUAN-CHIEHOstrowska, KingaChien, Shih-ChangOstrowski, StanisławChi-Feng, HungOtero, MartaChin, Young-WonOtsuka, YuzuruChinchilla, RafaelOuadi, AliChinnam, Parameswara RaoOuyang, LizhiChiorcea-Paquim, Ana-MariaOwczarek, AleksandraChira, KleopatraPacheco, MárioChirumbolo, SalvatorePaci, MaurizioChisholm, John D.Pacios, Luis F.Chitchumroonchokchai, ChureepornPadovan, AmandaChiu, Tai-chiaPaeng , Ki-JungChladek, GrzegorzPaganetti, PaoloChlichlia, KaterinaPagan-Torres, Yomaira J.Chmelová, DanielaPagay, VinayCho, Cheong-WeonPainter, GavinCho, Dong-WooPakulski, ZbigniewCho, EunaePalanisamy, Satheesh KumarCho, Hea-YoungPalazzo, EnzaCho, Nam-JoonPálinkó, IstvánCho, Somi KimPaliyath, GopinadhanCho, Ssang-GooPaller, ChanningChobot, VladimirPalmans, Anja R. A.Choi, HyukjaePalmieri, AlessandroChoi, Hyung-KyoonPalomino, Olga MaríaChoi, Jae SuePalomo, JoseChoi, Ki ChoonPalugan, LucaChoi, Nag JungPalumbo, CarlaChoi, Seok KiPan, Min-HsiungChoi, YoungPanaro, Maria AntoniettaChoi, Young HeePandey, SudipChoi, Young WhanPane, SalvadorCholewinski, GrzegorzPaneth, AgataChollet-Krugler, MarylènePanguluri, Siva KumarChong, Lee-GauPantazaki, Anastasia A.Chopp, MichaelPanzella, LuciaChopra, MridulaPaoli, PaolaChoshi, TominariPaoli, PaoloChou, C. JamesPaolo, ArosioChou, Cheng-YangPapadakis, RaffaelloChou, Kuo-ChenPapadimitriou, Sofia A.Chow, Simon Kwoon-HoPapadimitriou, VassilikiChriste, Karl O.Papageorgiou, George Z.Christensen, Jørn B.Papaioannou, DionissiosChristensen, LarsPapanastasiou, IoannisChristoffersen, ChristinaPapetti, AdeleChristoforides, EliasPapi, MassimilianoChristopher W., KirbyPapo, NivChristova-Bagdassarian, ValentinaPapoutsis, KonstantinosChrobok, AnnaPapp, AndrásChruszcz, MaksymilianPappas, ChristosChu, Ching-LiangPappas, JaniceChu, DafengParfenova, LyudmilaChu, JiaxiangParisini, EmilioChu, ShidongPark, Byung-DaeChu, Tin-ChunPark, Chan HeeChuang, Ta-hsienPark, Chan HumChueh, Pin JuPark, Dae-HunChun, Byung-sooPark, Hyung-hoChung, ChungwookPark, Il-KwonChung, DonghoonPark, In-KyuChung, HoyongPark, Jeong HoChung, Jing-GungPark, Jong-SeokChung, Man-KyoPark, Myung HwanChung, Tai-ShungPark, Seung-ChunChung, Ying-ChienPark, WansuChurcher, IanParkar, Shanthi G.Chuyen, Hoang V.Parkinson, Don-RogerChyan, Chia- LinParks, RobinChyau, Charng-CherngParpinello, Giuseppina PaolaChylewska, AgnieszkaParra, RobertoCiancaleoni, GianlucaParra-Guillen, Zinnia PatriciaCiardelli, FrancescoParrino, BarbaraÇiçek, Serhat SezaiParsons, J.G.Cicero, ArrigoPascual-Teresa, SoniaCichero, ElenaPasini, DarioCidade, HonorinaPasqua, GabriellaCież, DariuszPassalaqua, KarlaCigáň, MarekPassamonti, SabinaCimanga, KanyangaPassarella, DanieleCinar, ZekiyePastor, Isidro M.Ciobica, AlinPasz, Lidia SasCiofani, GianniPatel, HardikkumarCiregia, FedericaPatenaude, MathewCirillo, GiuseppePatrice, ThierryCísarová, MiroslavaPattanaik, HimansuCisowsky, JaroslawPatterson, Adam V.Citores, LuciaPatterson, JenniferCiulli, AlessioPauer, WernerCiulu, MarcoPaul, ThornalleyCiz, MilanPaulsen, Berit SmestadČizmić, MirtaPaululat, ThomasClark, AndrewPaulusse, JosClarke, Ronald JPautz, AndreaClaude, JulienPavan, FernandoClaus, HaraldPavão, Mauro S. G.Clerico, AldoPavela, RomanCoan, P. M.Pavic, ValentinaCobbold, SimonPavlidis, IoannisCocetta, GiacomoPavon-Djavid, GracielaCock, Ian EdwinPawełczyk, AnnaCoelho, José APawełkowicz, Magdalena EwaCoelho, PauloPaz, YaronCohen, SmadarPazdro, RobertColacino, EvelinaPazourek, JiriColdea, TeodoraPeana, Massimiliano FColecchia, DavidPeczuh, MarkColeman, Anthony WilliamPedatella, SilvanaColeman, William B.Pedersen, AndersColetta, MassimoPedeux, RémyColey, Helen M.Pedretti, AlessandroColin, YannickPedrosa, RafaelColl, JosepPedroso, EnriqueCollina, SimonaPeifer, ChristianCollins, Stuart G.Peigneur, SteveCologna, StephaniePelagalli, AlessandraColonna, GiovanniPellegrino, FrancescoColquhoun, Thomas A.Pellegrino, SaraColucci, GabriellaPellice, SergioComai, StefanoPellicer, EvaCombès, AudreyPellis, AlessandroComi, CristoforoPeluso, IlariaComito, Robert JPeña, Maria Marjorette O.Commane, DanielPence, BrandtCompadre, CesarPendleton, PhillipConcilio, SimonaPeng, Chi ChungCondello, MariaPeng, Chien-fangConlon, John MichaelPeng, RobertConstantinescu, StefanPeng, RuoguContino, MarialessandraPeng, TengConway, BarbaraPeng, Wen-HuangCook, Atlanta G.Penissi, Alicia B.Cook, Gregory R.Pennock, NathanCooper, Arthur J.L.Pentzold, StefanCooper, RolandPepeu, GiancarloCoote, PeterPeral, JoséCopetta, AndreaPerego, CarlaCopolovici, Dana MariaPereira, DavidCorazzin, MircoPereira, FlorbelaCorbi, GraziamariaPereira, José AugustoCorchado, Jose C.Pereira, LucianaCorchete, PurificacionPereira, LuizCorcione, Carola EspositoPereira, Maria Do CarmoCorke, HaroldPereira, Olívia R.Correa Pacheco, Zormy NacaryPereira, VandaCorreia, IlidioPerepelyuk, MarynaCorreia, Ilídio J.Perera, RushikaCorreia-de-Sá, PauloPerestrelo, RosaCorrigan, DamionPerez Nevado, FranciscoCossio, FernandoPérez, Andy J.Costa Pessela João, BenevidesPerez, JaraCosta, Blaise MathiasPerez, LarkCosta, GiosuèPérez, SilviaCosta, Maria-margarida R. R.Pérez-Castrillón, José L.Costa, RosariaPérez-Díaz, I.M.Costa, RuiPerez-Guaita, DavidCosta, Susana P.G.Perez-Stable, CarlosCostantino, LucaPerez-Vizcaino, FranciscoCostantino, ValeriaPerić, MihaelaCosti, Maria PaolaPerinelli, Diego RomanoCotea, Valeriu V.Perna, SimoneCouce, María D.Perosa, AlviseCouch, RobinPerrais, MichaëlCourty, JoséPerrio, CecileCoutinho, João PauloPerrone, GiancarloCoutsolelos, AthanassiosPersico, MarcoCoyne, Anthony G.Pertusati, FabrizioCrabtree, Robert H.Pescitelli, GennaroCragg, PeterPestov, AlexandrCranfield, CharlesPeter, MartinCrespo, IrenePéterfy, MiklósCrișan, GianinaPeterson, JuliaCrocket, KirstyPeterson, LarrynCrowley, James D.Petit, MarcCsuk, RenePetit, Patrice X.Cui, GanglongPetrini, MarinoCui, MengchaoPetropoulos, SpyridonCunha, AngelaPetrov, ViacheslavCunha, RodrigoPetrova, Krasimira T.Cunningham, Jeffrey A.Petrová, ŠárkaCuny, Gregory D.Petta, IoannaCurti, ClaudioPetzer, Jacobus P.Czarnocki, ZbigniewPeyton, David H.Czekelius, ConstantinPfeffer, Frederick M.Czerwinska, MonikaPhilipose, U.Czyżowska, AgataPhillips, Ana Maria Madeira Martins FaíscaD. Skaltsa, HelenPhillips, DonD’Aiuto, LeonardoPhillips, Robert S.Da Paixão, Thiago Regis Longo CesarPhylactou, Leonidas A.Da Silva, Joaquim C.G.E.Piao, XijunDa Silva, José P.Piazza, Gary A.Dabrowski, JanuszPiccoli, GBDacarro, GiacomoPicha, JanD’accolti, LuciaPicone, GianfrancoDada, LauraPieczykolan, BarbaraDagorne, SamuelPiedade, Ana PaulaD’Agostino, NunzioPiemontese, LucaDagvadorj, JargalsaikhanPierelli, LucaDai, YuminPieroni, MarcoDai, ZiweiPierre, GuillaumeDaiguebonne, CarolePieters, LucDal Monte, MassimoPieters, RolandDaley, PeterPietrella, DonatellaDall’Acqua, StefanoPigge, F. ChristopherDalmo, Roy A.Pignatello, Joseph JDalton, DavidPignitter, MarcDaly, NorellePillaiyar, ThanigaimalaiDamjanovski, SashkoPillay, VinessDamkaci, FehmiPimenta, Daniel CarvalhoDa’na, EnshirahPIN, Jean-MathieuDaniel, Marie-ChristinePinazo, AuroraDanielak, DorotaPindi, SureshDanielson, HelenaPineda, MiguelDaniels-Wells, Tracy R.Pinnapireddy, Shashank ReddyDansette, Patrick M.Piñol, MilagrosDarvesh, SultanPinto, Diana C. G. A.Das, Diganta BhusanPinto, MafaldaDas, SaurabhPinu, Farhana R.Das, ViswanathPinzaru, IuliaDasari, RameshPiotrowska, UrszulaDasgupta, KasturiPiriou, YannickDash, Alekha K.Pirman, TatjanaDaszykowski, MichalPisarcik, MartinD’Auria, MaurizioPistone, AlessandroDaverey, AmitaPitucha, MonikaDavid, LuminiţaPiva, TerrenceDavies, KevinPiwowar, AgnieszkaDavioud-Charvet, ElisabethPlanas, MartaDavis, BurtronPlano, DanielDawadi, SurendraPlantinga, TheoDe Agostini, ArianePla-Quintana, AnnaDe Almeida Coelho De Sousa, Maria JoãoPlastina, PierluigiDe Baerdemaeker, TreesPlatas-Iglesias, CarlosDe Bairros, André VallePlatts, James A.De Bellis, LuigiPlaza, AlbertoDe Berardis, DomenicoPlaza-Díaz, JulioDe Caro, VivianaPlażuk, DamianDe Castro, SoniaPlemper, Richard K.De Falco, EnricaPlesiat, PatrickDe Feo, VincenzoPlourde, Guy L.De Geest, BartPlutino, Maria RosariaDe Jonghe, StevenPoddel’sky, AndreyDe Kimpe, NorbertPoetsch, AnsgarDe La Garza, MireyaPogorzelska, AnetaDe La Luz Garcia-Hernandez, MariaPohl, EhmkeDe La Mata, Francisco JavierPoinsot, VerenaDe La Prida, Victor ManuelPoisson, Jean-FrançoisDe Lourdes Pereira, MariaPolasek, Thomas M.De Marco, RossellaPolesel, FabioDe Martino, Maria GabriellaPolet, MichaëlDe Mendonça, Dina I. M. D.Poli, GuidoDe Menezes, Irwin RoseAlencarPolischuk, PavelDe Oliveira, Luiz Fernando CPollmann, KatrinDe Paola, IvanPomerantz, William C. K.De Pauw, EdwinPons, JosefinaDe Paz, Jose LuisPontalier, Pierre-YvesDe Riccardis, FrancescoPontiki, EleniDe Ruyck, JérômePoór, Miklósde Sousa, Maria EmíliaPopat, AmiraliDe Souza, Maria De Fátima VanderelyPopa-Wagner, AurelDe Spiegeleer, BartPopielarz, RomanDe Vendittis, EmmanuelePopiołek, ŁukaszDe Vries, MattanjahPoplawski, TomaszDe Wit, MarynaPopov, Alexey A.Deev, Sergey L.Popov, AnatoliyDeevska, GerganaPopović Đorđević, JelenaDegano, IlariaPopovich, DavidDehaen, WimPoriel, CyrilDehghan, EsmailPorro, ChiaraDel Corso, AntonellaPorter, Brenda E.Del Giudice, RitaPotapenko, Oleg V.Del Rio Celestino, MercedesPotapov, AndreiDel Rosal, BlancaPotterat, OlivierDelattre, CédricPoucheret, PatrickDelbarre-Ladrat, ChristinePouli, NicoleDelbecq, FredericPower, PhilipDelfine, SebastianoPowers, EvanDeligios, Paola A.Powers, RobertDella Ca’, NicolaPoynter, Matthew E.Della Ciana, LeopoldoPozo, Óscar J.Della Sala, GerardoPrasad, SahdeoDelogu, FrancescoPrassl, RuthDelorme, VincentPratsinis, HarrisDembska, Anna RenataPrehna, GerdDemchenko, Alexei V.Prentice, HowardDemirhan, ErhanPrescott, John F.Demopoulos, Vassilis J.Price, Joshua L.Den Hertog, JeroenPrice, Neil P. J.Denat, FranckPrieto, MariaDeng, JingPrigent, SylvainDeng, PengPrima-García, HelenaDeng, Win-PingProenca, FDeNicola, GinaProestos, CharalamposDenny, BillProft, ThomasDeo, PermalProserpio, DavideDeo, RandhirProsser, Robert ScottDeplazes, EvelyneProtti, MicheleDeriu, Marco AgostinoProvinciali, MauroDeRosa, MariaPruvost, AlainDerosa, PedroPrzybylski, PiotrDerossi, AntonioPsomas, GeorgeDesai, Umesh R.Psurski, MateuszDeslouches, BerthonyPucci, AndreaDesmaële, DidierPuga, Alberto V.Desmarets, ChristophePuglia, CarmeloDesmet, TomPuglisi, FabioDessolin, JeanPunchard, Neville ADeutsch, EmericPunganuru, Surendra ReddyDey, GangotriPunzo, FrancescoDhakshinamoorthy, AmarajothiPuri, AkshitDhanasekaran, Danny N.Püschel, Gerhard P.Dharavath, SrinivasPushkar, YuliaDi Gioia, Maria LuisaPusztahelyi, TündeDi Santo, RobertoPutnik, PredragDi, LiPutt, KarsonDiaba, FaïzaPutz, MihaiDiamanti, Maria VittoriaPy, SandrineDiana, PatriziaPyka-Pająk, AlinaDias, JulianaPyta, KrystianDias, NicolinaQaroush, AbdussalamDiaz, GasparQi, GuangyanDibble, PeterQi, Hong-LanDichiara, Anthony BQian, Bing-JunDickert, Franz L.Qin, HailinDickinson, ElizabethQiu, HongdengDidonna, AlessandroQiu, LipingDiekmann, JanQiu, Ming-huaDietzel, Pascal D.C.Quadrelli, PaoloDíez, DavidQueiroga, Carmen LuciaDikalov, SergeyQuesada-Cabrera, RaúlDillert, RalfQuijada-Garrido, IsabelDilman, Alexander D.Quiles, José L.Dimri, GoberdhanQuiliano, MiguelDing, BaoqingQuinn, Mark T.Dini, DaniloQuintana, JoséDinnebier, RobertQuintavalla, AriannaDionisio, GiuseppeQuirino, Joselito P.Dixon, David A.Qvit, NirDoda, Sai ReddyR. Odell, LukeDoh, Kyung-OhRaabe, GerhardDoi, HisashiRabanal, FrancescDoi, TakayukiRabenstein, Dallas L.Dolzhenko, Anton V.Rachwalski, MichałDomcke, WolfgangRadic, ZoranDomínguez Alvarez, EnriqueRadwan, Mohamed M.Domínguez-Álvarez, EnriqueRaes, KatleenDonato, LauraRagno, RinoDonato, RosarioRahimi, FaridDong, LanfengRahman, MasudurDong, MingdongRahme, KamilDong, ShihuiRai, Dilip K.Donnio, BertrandRai, GaneshaDonno, DarioRai, PrakashDonzelli, ElisabettaRaimondi, LaviniaDoppalapudi, Rupa S.Rais, RanaDore, Timothy M.Raj, Madhwa H.G.Doria, FilippoRaja, R.Dorin, JuliaRajauria, GauravDormidontova, ElenaRaje, MithunDorta, RetoRajendran, PraveenDos Santos, José C.S.Rajić, NevenkaDosio, FrancoRajnarayanan, RajendramDosoky, Noura S.Rakesh, PuttreddyDotsikas, YannisRakitin, OlegDouroumis, DennisRaliya, RameshDovinova, ImaRamachandran, AnupDrabowicz, JózefRamaiahgari, Sreenivasa C.Drain, PeterRamakrishnan, SrinivasanDreiser, JanRamalho, TeodoricoDrlica, KarlRamalhosa, ElsaDrosinos, EleftheriosRamamoorthy, AyyalusamyDrougard, AnneRamasamy, Uma ShantiniDruzbicki, KacperRambla, José L.Du, PufengRamis, GianguidoDu, ShushanRamkumar, VickramDu, YongRamona, PaltineanDuarte, AndreiaRamos, Adrián M.Duba, KurabachewRamos, SoniaDudonné, StéphanieRamos-Diaz, Jose MartinDüfer, MartinaRamos-Ortiz, GabrielDufossé, LaurentRamsay, RonaDugé De Bernonville, ThomasRangnekar, AbhijitDughera, StefanoRanzato, EliaDugo, GiacomoRapeanu, GabrielaDuh, Chang-YihRaposo, Maria Filomena De JesusDuh, Pin-DerRapposelli, SimonaDuhamel, JeanRaptis, Raphael G.Duke, Stephen O.Rasapalli, SivappaDumur, FrédéricRasmussen, HelgeDundas, IanRasmussen, Silas AnselmDunietz, Barry D.Raspoli, AnnaDuong, Hai MRateb, Mostafa E.Dupont, NathalieRatkova, Ekaterina L.Dupureur, Cynthia M.Ratova, MarinaDurap, FeyyazRatovitski, EdwardDurazzo, AlessandraRatz-Łyko, AnnaDurek, ThomasRaucci, MgDurgo, KsenijaRaupp, Sebastian M.Dussault, PatrickRaut, SangramDutta, ArijitRautio, JarkkoDuval, Raphael E.Rauwel, ProtimaDuverger, EricRavegnini, GloriaDvoyashkin, MuslimRawel, HarshadraiDwivedi, ChandradharRay Dias, JerryDykman, Lev A.Ray, SwayamjitDzade, NelsonReaney, Martin J. T.Dzida, KatarzynaReany, OferDziubla, ThomasRebollar, EstherDzmitry H., ZaitsauRedaelli, SaraDzyuba, SergeiReddy, SakamuriEasmon, JohnnyRedshaw, CarlEaton, PeterReese, R. NeilEberini, IvanoReeve, VivienneEbihara, LisaRegal, PatriciaEblagon, KatarzynaRehder, DieterEbrahim, WeaamRehman, Junaid U.Echevarria, JuanRehrig, Erin M.Eckenhoff, Roderic G.Reina, MEckl, PeterReinaud, OliviaEdafiogho, IvanReinecke, HelmutEddy, NicholasReisman, Scott A.Edin, Nina JeppesenReissmann, SiegmundEdrada-Ebel, RuAngelieReiter, RusselEeckhaut, Ann VanRekka, Eleni A.Efferth, ThomasRen, DongmeiEfstathopoulos, Efstathios P.Ren, JianguoEgami, HiromichiRen, ShuxinEgberts, PhilipRen, YulinEggertsen, GostaRenard, Pierre-YvesEhara, MasahiroRencoret, JorgeEhm, ChristianRendina, Louis M.Ehrich, MarionRendón Huerta, Erika PatriciaEisenreich, WolfgangRenou, MichelEkiert, HalinaRescifina, AntonioEklund, Patrik C.Resende Maldonado , RafaelEkström, FredrikReta, MarioEkuni, DaisukeReuter, KlausEl Kaïm, LaurentReverberi, MassimoElameen, AbdelhameedRevilla, EugenioEldeeb, MohamedReyes Parada, MiguelElemans, Johannes A.A.W.Reyes, FernandoElia, Angela RitaReyes-Chilpa, RicardoElkins, KellyReynisson, JóhannesEller, FredRezzani, RitaElli, StefanoRho, Jung-RaeEl-Safty, Sherif ARiccardo, IentileElse, KathrynRiccardo, Zenezini ChiozziEl-Seedi, Hesham R.Riccetti, LauraElsinghorst, Paul W.Ricci, AriannaEmpting, MartinRice, HelenEnchev, VenelinRicelli, AlessandraEndres, KristinaRicha, TambiEngel, JasperRichardson, AlanEngh, Richard A.Richomme, PascalEnriquez, Raul G.Riedl, RainerEpelde Bejerano, EvaRigano, DanielaEppard, ElisabethRigano, Dott. DanielaErb, WilliamRigano, Maria ManuelaEritja, RamonRijo, PatríciaErxleben, AndreaRimawi, Fuad AlEsatbeyoglu, TubaRimer, MendellEscalante García, JaimeRimez, BartEscames, GermaineRimondini, LiaEscario, JoséRinnerthaler, GabrielEspartero, José L.Rios-Torres, RamonEsplugas, SantiagoRischer, HeikoEsposito, Carla L.Riscuta, GabrielaEsposto, SoniaRishi, ArunEstelrich, JoanRitter, HelmutEsteve-Romero, JosepRivas, FatimaEstevez, Jorge D.Rivero-Pérez, M. DoloresEstevez, Ramón J.Rivière, CélineEstour, FrançoisRobberecht, HarryEto, BrunoRoberto, ConsonniEuaggelos, GikasRoberts, SigridEvanno, LaurentRoberts, Sue A.Evtugyn, GennadyRobertson, Mark J.Fabiani, RobertoRobien, WolfgangFabio Zuluaga, HectorRobiette, RaphaelFacchiano, AngeloRobles Medina, AlfonsoFacchin Muñoz, GianellaRoby, Mohamed Hussein HamdyFaenza, IreneRoca-Sanjuán, DanielFaggio, CaterinaRocchetti, GabrieleFahlman, Richard P.Rocchia, WalterFaísca, PatríciaRocchitta, GaiaFajardo, MarianoRochais, ChristopheFajín, José L. C.Roche, Daniel BarryFakhoury, AhmadRodrigues-Santos, Claudio E.Fakis, MihalisRodríguez Sanoja, RominaFalanga, AnnaritaRodriguez, Hortensia M.Falletta, ErmelindaRodríguez, Jesús ÁlvarezFan, ChenguangRodríguez, LauraFan, LiRodríguez, MarioFan, YunchangRodriguez-Castellon, EnriqueFan, ZhijinRodríguez-López, JuliánFanfrlik, JindrichRodriguez-Mateos, AnaFanfrlík, JindřichRoelants, KimFang, BaiRogers, SimonFang, Li-WenRogozhin, EugeneFanizzi, Francesco P.Roh, ChanghyunFantacuzzi, MarialuigiaRoh, JaroslavFaraldos, MarisolRoh, SanghoFardel, OlivierRoivainen, AnneFarhat, GraceRojas, JanneFarinha, CarlosRokstad, Anne MariFarkas, ÁgnesRoman, JesseFarris, StefanoRomano, PatriziaFarrugia, BrookeRomee, RizwanFasinu, Pius S.Romero, AldoFasulo, SalvatoreRomero, IsabelFaustino, Maria AmparoRomieu, AnthonyFauzi, IlianaRonca, SaraFava, EugenioRoot, Martin M.Favre-Réguillon, A.Ropiak, Honorata M.Feas, XesusRos García, José MaríaFedorova, Olga V.Röschenthaler, Gerd-VolkerFeliu, LidiaRosenberger, Thad A.Feng, Wen-HaiRosenberry, TerroneFeng, YuRosendahl, JonasFenical, WilliamRosendale, Douglas IanFeo, FrancescoRosés, MartíFeresin, Rafaela G.Rosi, MarzioFernandes, AntonyRosini, MichelaFernandes, Caio PinhoRoss, KellyFernandes, CarlaRossi, DanielaFernandes, P.Rossi, MiriamFernandes, PedroRostagno, Mauricio ArielFernandes-Ferreira, ManuelRota, Paul A.Fernández García, ElisabetRoterman, IrenaFernandéz, CarlosRouse, RodneyFernández, InmaculadaRoussel, ChristianFernández, MartaRoutray, WinnyFernández, RobertoRovero, PaoloFernández-Ballester, GregorioRowinska, MagdalenaFerrante, ClaudioRoy, BimolFerreira, JorgeRoychowdhury, SanjoyFerreira, Leonardo G.Różalska, BarbaraFerreira, Marcelo José PenaRozalski, MarcinFerrieres, VincentRozen, ShlomoFeussi Tala, MichelRozhon, WilfriedFigadere, BrunoRtimi, SamiFigueiral, Maria HelenaRuan, Chang-ChunFigueiredo, Ana Cristina SRuan, KeFilarowski, AleksanderRubio-Arraez, S.Filippi, Jean-jacquesRubiolo, Pat6riziaFilippov, AnatolyRudick, JonathanFina, EmanuelaRudyk, OlenaFinley, JohnRüegg, Urs T.Finšgar, MatjažRuff, AdrianFiore, AlbertoRuggerone, PaoloFirer, MichaelRuggieri, FabrizioFirlej, LucynaRugman-Jones, PaulFischer, SusannRühl, MartinFisher, P.B.Ruiu, LucaFiszer, AgnieszkaRuiz-Aceituno, LauraFlanjak, IvanaRuiz-Gómez, GloriaFleischer, IvanaRulíšek, LubomírFleury, YannickRusmini, PaolaFlieger, JolantaRussell, Fraser D.Florczyk, Stephen J.Russo, Gian LuigiFlower, DarrenRusso, MariaFochi, MariafrancescaRusso, MarinaFodor, CsabaRusso, NinoFodor, MariettaRustin, PierreFontés, MichelRuthstein, SharonFoo, EloiseRuzza, PaoloForbes-Hernández, TY.Ryabchun, AlexanderForlani, GiuseppeRyan, JackForli, StefanoRyan, MichaelFormisano, CarmenRybak, LeonardFormisano, LuigiRyu, Jong HoonForner, StefâniaRyu, Shi YongForneris, FedericoRyu, Stephen BeungtaeFossen, TorgilsRyu, Sung KeeFosso, MarinaRzepecka-Stojko, AnnaFossum, EricSa, JacintoFoubelo, FranciscoSacco, AdrianoFracassetti, DanielaSacco, PasqualeFraile, AlbertoSachs, GeorgeFraile, José M.Sackus, AlgirdasFranca, Eduardo FSadovskaya, IrinaFranchini, CarloSadowski, LukaszFrancisco, Ana PaulaSaeed, Mohamed ElfatihFranco, LuisSaenz-Mendez, PatriciaFranco, RodrigoSáez, EduardoFrancolini, IolandaSagui, CelesteFrank, SašaSaha, AchintoFrau, JuanSaha, MrinmoyFrau, RobertoSahariah, PriyankaFreakley, Simon J.Saiano, FilippoFreccero, MauroSaid, Ahmed M.Freitas, AnaSaielli, GiacomoFreitas, Ana C.Saigusa, DaisukeFribley, Andrew M.Sainlos, MatthieuFrick, DavidSaisho, YoshifumiFröde, Tânia SilviaSaitoh, KenjiFroeyen, MatheusSakamoto, TakeharuFröhlich, EleonoreSakilam, SatishFrolova, LiliyaSakumura, YuichiFrongia, AngeloSala, RobertoFrontera, AntonioSalaheen, SerajusFruhmann, PhilippSalahub, DennisFruit, CorinneSalazar-Olivo, LuisFrungillo, LucasSaleem, MuhammadFrye, RichardSaleh, MahmoudFu, JunjieSalerno, LoredanaFu, LiSalim, VonnyFuhrer, Timothy J.Sallem-Idrissi, NaimaFujihara, HisakoSalmerón, IvanFujihara, TetsuakiSalomone, FedericoFujimori, KoSalopek-Sondi, BrankaFujisawa, TomotsumiSalud Bea, Roberto De LaFujita, MasakiSaluzzo, ChristineFujita, MikakoSalvo, AndreaFujita, MorifumiSamanidou, VictoriaFukuda, TakeshiŠamec, DunjaFukunaga, KenjiSampaio, AnaFukushi, KeiichiSamuels, IvyFukushima, AtsushiSan Martin, CarmenFuller, AmeliaSanchez, Diego H.Fullerton, MorganSanchez, GoarFunel, NiccolaSánchez-Barceló, EmilioFurkert, Daniel PlimmerSánchez-Fidalgo, SusanaFürnsinn, ClemensSánchez-García, DavidFurukawa, YoshiakiSanchez-Madrid, FranciscoFurusawa, ChikaraSánchez-Moreno, ManuelFuruta, HiroyukiSánchez-Pérez, RaquelG. Lago, João HenriqueSanchis, M.J.Gadzovska Simic, SonjaSancho, JaimeGaeta, CarmineSancho, TeresaGăină, Luiza IoanaSancineto, LucaGajdziok, JanSandhu, Jagdeep K.Gál, PeterSandjo, Louis PergaudGalamba Correia, João DomingosSang, ShengminGalanis, AlexSanganyado, EdmondGalano, AnniaSangaraju, DewakarGalan-Vidal, Carlos AndresSangiovanni, EnricoGalat, AndrzejSanjust, EnricoGalati, FrancescoSankaran, Ganapathy SubramanianGale, EricSankaranarayanan, Nehru VijiGaleazzi, RobertaSannicolò, FrancescoGales, LuísSanoh, SeigoGalgonek, JakubSansone, ClementinaGaliano, SilviaSanter, SvetlanaGall Troselj, KoraljkaSantilio, AngelaGallo, CarmelaSantillo, MichaelGallo, GiuseppeSantin, Silvana M.O.Galván-D’Alessandro, LeandroSantini, AntonelloGalzitskaya, Oxana V.Santos, ClementinaGan, RenyouSantos, Maria M. M.Ganesan, A.Santos, SusanaGao, FengSapino, SimonaGao, JunSarabia, FranciscoGao, WenyunSardon, HaritzGao, ZheSarkar, DhrubaGao, ZhiqiangSarrou, EiriniGarcía Álvarez, JoaquínSasai, YasushiGarcía Viguera, CristinaSassi, YassineGarcia, Coralia V.Sastre, EnriqueGarcia, Jose M.Satake, MasayukiGarcía-Fresnadillo, DavidSato, HisakoGarcía-Frutos, Eva MaríaSato, KenjiGarcía-Garrido, Sergio E.Sato, ShinGarcía-Ortega, LucíaSatoh, ShigeruGarcia-Sosa, Alfonso T.Satou, TadaakiGarcía-Villalón, Angel LuisSatriano, CristinaGargiulo, SimonaSaturnino, CarmelaGarinis, GeorgeSatyal, PrabodhGarno, JayneSaurina, JavierGarrote, GilSavi, MoniaGasparrini, MassimilianoSaviano, MicheleGaumann, AndreasSavoie, Jean-MichelGavara, LaurentSavy, DavideGawel, RichardSawai, HirofumiGdaniec, ZofiaSawicki, RafalGe, XiaoweiSayre, Casey L.Ge, YubinSbardella, GianlucaGellerman, GaryScalici, TommasoGendaszewska-Darmach, EdytaScalise, MariafrancescaGendron, Fernand-PierreScanlon, Martin J.Genta-Jouve, GrégoryScarso, AlessandroGentilucci, LucaScavello, FrancescoGenuino, Homer C.Schaberle, Till F.Georghiou, P.Schaller, G. EricGeorgiades, SavvasSchaller, HubertGeorgiadi, AnastasiaSchang, LuisGeorgiev, VasilSchebb, NilsGeppi, MarcoSchenone *, SilviaGerbier, PhilippeSchepdael, Ann VanGeremia, SilvanoSchepetkin, Igor AGerke, JörgScherer, GüntherGerner, ChristopherScherf, KatharinaGeronikaki, AthinaScherf, UllrichGerothanassis, Ioannis P.Schieber, AndreasGeso, MoshiSchinnerl, JohannGeu Flores, FernandoSchisler, JonathanGhaste, ManojSchlitzer, MartinGhatak, ArindamSchmeda Hirschmann, GuillermoGherardi, FrancescaSchmid, J.Ghosh, AnamitraSchmidt, SandyGhosh, SantoshSchmidtke, Leigh M.Giaginis, CostantinosSchmit, JeremyGiamperi, LauraSchmitz, John C.Giampieri, FrancescaSchnackenberg, LauraGiancane, GabrieleSchneider, BerndGiannakas, ArisSchneider, Elena KatharinaGiannini, CleliaSchneider, RaphaelGiannoni, PatriziaSchnepf, AndreasGiaouris, EfstathiosSchoner, WilhelmGiarratana, FilippoSchonthal, AxelGibert, YannSchopfer, Lawrence MGiddings, Lesley-AnnSchörken, UlrichGil, AntonioSchott, M.Gil, DmitrySchouten, AlexanderGilch, SabineSchrader, AndreaGiménez, RaquelSchrag, MatthewGimeno, M. ConcepciónSchrenk, MatthewGiordano, CristinaSchröder, CarolaGiotta, LiviaSchroeckh, VolkerGiovarelli, MirellaSchroeder, GrzegorzGirlando, AlbertoSchuehly, WolfgangGiuffrè, Angelo MariaSchulze, JohannesGlaubitz, ClemensSchurig, VolkerGloria, AntonioSChwalbe, Carl H.Gnavi, GiorgioSchwan, MarinaGobin, Andrea S.Schwan, William R.Goettig, PeterSchwarze, MichaelGoga, MichalSchweitzer, DietrichGoh, Boon ChongSchwikkard, SianneGohar, EmanŚcianowski, JacekGohda, EiichiScognamiglio, MonicaGoicoechea, NievesScott, PeterGolczak, MarcinScotti, MarcusGold, BenScovassi, Anna IvanaGoldsmith, Chloe D.Scozzafava, AndreaGoldstein, Peter AScudiero, OlgaGoldup, StephenSeca, AnaGolovanov, Alexander P.Seca, Ana M. L.Golubkina, Nadezda AleksandrovnaSecci, DanielaGombart, AdrianSecu, MihailGomes, Ana P.Sedghizadeh, ParishGomes, AndreiaSegarra-Marti, JavierGomes, Nelson G.M.Seijas Vázquez, JulioGomes, SóniaSeiner, Brienne N.Gómez De Cedrón, MartaSeiquer, IsabelGómez, Ana MaríaSejong, OhGomez, M. VictoriaSekimizu, KazuhisaGomez, NidiaSellner, JohannGomez-Garcia, CarlosSellstedt, MagnusGómez-Jeria, Juan S.Selvaggini, RobertoGómez-Sala, BeatrizSen, UtpalGoncalves, Luis MoreiraSendker, JandirkGonçalves, M. Sameiro T.Sendón, RaquelGonda, SándorSengupta, BidishaGondhalekar, Ameya DSeo, Jae HongGondi, Christopher SSeo, Jung-KilGong, Young-DaeSeo, Woo DuckGóngora-Castillo, ElsaSeoud, Omar ElGontier, EricSepčić, KristinaGonzález Domínguez, RaúlSerafino, AnnaluciaGonzález, Florenci V.Sergeant, KjellGonzález, IsraelSergi, Consolato MariaGonzalez, JoaquinSerio, AnnalisaGonzález, SergioSerpe, LoredanaGonzalez, VictorSerpersu, Engin H.Gonzalez-Barreiro, CarmenSerra, DolorsGonzález-Burgos, Elena MariaSerra, StefanoGonzález-Díaz, HumbertoSerradji, NawalGonzález-Maeso, JavierSerralheiro, Maria L.González-Rodríguez, María-LuisaSerrano, MaríaGonzález-Stuart, ArmandoSerrão, José EduardoGonzález-Vergara, EnriqueSethi, GautamGorb, L.Seto, ShigekiGoreham, ReneeSetyawati, Magdiel InggridGorgoulis, VassilisSetzer, William N.Gori, AntonellaSferruzzi Perri, AmandaGorini Da Veiga, AnaShaaban, Khaled AGorinstein, ShelaShah, DilipGorman, GregShah, ZahoorGorobets, NikolayShahar Yar, MohammadGórska, SabinaShahraki, MehdiGoryacheva, IrinaShakya, Viplendra Pratap SinghGośliński, TomaszSham, Yuk YGossage, R. A.Shang, ZhuoGosselet, FabienShanmugam, MuruganandanGotor-Fernández, VicenteShapiro, Bruce A.Götte, MartinShapter, Joe G.Gouany, FradySharma, AnjaliGoulas, VlasiosSharma, Arun KGoulay, FabienSharma, AtulGouws, ChrisnaSharma, BheshGozzo, FrancoSharma, Piyush SindhuGrabowski, SławomirShatkay, HagitGraier, WolfgangShavandi, AminGraiff, ClaudiaShavrukov, YuriGranados-Principal, SergioShaw, SubrataGrande, FedoraShehu, AmardaGranet, RobertShen, QilongGranica, SebastianShen, Yue-MaoGrant, GeorgeShen, ZhiqiangGras, EmmanuelShenderovich, Ilya G.Grassi, AlfonsoSheng, RongGraton, JérômeSheremetev, Aleksei B.Gravel, EdmondSherwood, JamesGrayer, Renée J.Sheu, Jyh-HorngGreco, AntonioShi, DaxinGreen, IvanShi, HaitaoGreenberg, Robert M.Shi, HuaGreenberger, Joel S.Shi, Jie-huaGreene, Catherine M.Shi, MichaelGregorio, Rosa PerezShi, QiangGrembecka, MałgorzataShi, YujunGribble, GordonShi, ZhenzhenGriesbeck, AxelShia, Kak-shanGrignani, GiovanniShiao, S. Pamela K.Grilli, SeleneShiao, Young-JiGrimm, Marcus O. W.Shibano, MakioGrindley, T. BruceShibata, NorioGrisanti, LucaShibayama, NobukoGrkovic, Dr. TanjaShieh, Tzong-MingGros, Claude P.Shim, JiwookGros, FrédéricShim, Yhong-heeGrossi, GiancarloShimizu, YoheiGrossini, ElenaShimodaira, ShigetakaGrosso, ClaraShin, Beom SooGroundwater, Paul W.Shinada, TetsuroGruber, JanShinomiya, KazufusaGruhlke, MartinShiono, TakeshiGruľová, DanielaShiragami, TsutomuGruszecki, WieslawShityakov, SergeyGryko, DanielShoeva, Olesya YuGrynkiewicz, GrzegorzShokouhimehr, MohammadrezaGrzebelus, DariuszShou, DanGu, ChengShpigelman, AviGu, ZhenShu, Hung-YeeGuallar, VictorShukla, ShrutiGuerrero, AntonioShukla, Sudheesh K.Guerrini, AlessandraShults, Elvira E.Guerrini, MarcoShumyantseva, Victoria V.Guevara-Fefer, PatriciaShun, YaoGueven, NuriSiatka, TomášGuitou, MarieSiciliano, CarloGulgunje, PrabhakarSieber, FritzGullón, BeatrizSiegel, JakubGumbo, Jabulani R.Sienkiewicz, MonikaGumieniczek, AnnaSierecki, EmmaGumienna, MałgorzataSiewert, BiankaGunaje, JayaramaSignore, GiovanniGunia-Krzyżak, AgnieszkaSignoretto, MichelaGuo, FenghaiSilaghi-Dumitrescu, RaduGUO, PUSilman, IsraelGuo, ZhigangSilva, FilomenaGupta, SanjuSilva, LuísGurevich, VesovoldSilva, Luís R.Guriec, NathalieSilva, Márcio S.Gurley, Bill J.Silva, Maria FátimaGurramkonda, CHANDRASEKHARSilva, PedroGursoy, GamzeSilva, Susana N.Gustafson, KirkSilvério, Sara C.Gutierrez De Teran, HugoSilvi, BernardGutierrez, SantiagoSimha, RamanujaGutierrez-Gines, Maria JSimin, NatasaGutiérrez-Uribe, JanetSimirgiotis, Mario J.Gütschow, MichaelSimitzis, PanayiotisGuzik, UrszulaSimko, FedorGuzow-Krzemińska, BeataSimmonds, Monique S.J.Guzzo, FlaviaSimmons, TrevorH Van Der Horst, EdwardSimone, Angela DeH. Moreno, Paulo RobertoSimone, GiuseppinaHa, DonhyungSimonet, Ana M.Habata, YoichiSimpson, Julie E.Habila, MohamedSims, Ian M.Habuda-Stanic, MirnaSingh, AnilHachiya, AkiraSingh, AnirudhaHadad, ChristopherSingh, AshutoushHadjiargyrou, MichaelSingh, HarinderHadjikakou, SotirisSingh, PriyankaHadjikakou, Sotiris K.Singh, RajbirHaider, NorbertSingh, RanjeetHaiges, RalfSingh, SashaHajdari, AvniSingh, SomnathHalle, StephanSingh, Vijay P.Hallsworth, J.E.Singh, VishalHalmosi, RóbertSinha, AbhijeetHamaguchi, MasahideSiodmiak, TomaszHamman, SiasSipponen, Mika HenrikkiHamon, Jean-RenéSiracusa, LauraHampel, DanielaSiren, HeliHan, Dong WookSitarek, PrzemysławHan, JaehongSiwek, AgataHan, JianlinSizochenko, NataliaHan, Quan-BinSkalicka - Woźniak, KrystynaHan, Se JongSkalko-Basnet, NatasaHan, Zhengxu S.Skenderi, Katerina PHandge, Ulrich A.Skidmore, MarkHandzlik, JadwigaSkorik, Yury A.Hanhineva, KatiSlavik, RogerHanif, MuhammadSlavov, AntonHano, ChristopheSławiński, JarosławHansel, ColleenSledzinski, TomaszHansen, EspenSloan, Kenneth B.Hansen, NielsSłoczyńska, KarolinaHansen, Paul RobertSlominski, AndrzejHanus-Fajerska, EwaSmani, YounesHao, LongyunSmeds, Annika I.Haouas, MohamedŠmejkal, KarelHarasym, JoannaSmeriglio, AntonellaHarenberg, JSmirnov, Alex I.Harmat, VeronikaSmith, MarkHarpke, DörteSmithrud, DavidHarrison, Paul H. M.Snyder, ScottHaruki, MitsuruSoavi, FrancescaHasegawa, MorifumiSobeh, MansourHashimoto, MakotoSobkowski, MichalHashimoto, MasaruSobrado, PabloHashimoto, MasayukiSobral, Abilio J. F. N.Hashimoto, NaotoSoengas, RaquelHashizume, MineoSoeta, TakahikoHashmi, A. Stephen K.Sohn, Uy DongHashmi, IrinaSokół-Łętowska, AnnaHassan, Hassan M. A.Sokolsky, MarinaHassan, MohamedSolano, FranciscoHassan, Sherif T. S.Solcan, CarmenHatae, NoriyukiSoldevilla, MarioHatano, TsutomuSoler Aznar, MariaHatzakis, EmmanuelSolomon, MelaniHaufe, GünterSolomonov, BorisHaug, Bengt ErikSoloshonok, Vadim A.Hausner, GeorgSomani, SukrutHawes, Chris S.Sommerer, NicolasHawkridge, Adam M.Song, EdwardHayashi, ShotaroSong, JinhuaHe, DinggengSong, JinhuiHe, GuSong, KenanHe, HailunSong, Kwang HoHe, HongSong, LingHe, HongliangSong, Sang Hoonhe, yaningSong, WeihuaHearn, Michael J.Soon, AloysiusHeber, DieterSorensen, DanHeger, MichalSorensen, Darwin L.Heinrich, Markus R.Soto-Hernández, MarcosHeiskanen, JuhaSoulère, LaurentHeiss, ElkeSovova, HelenaHeld, ChristophSpanakis, MariosHellens, RogerSpano, GiuseppeHelm, RichardSpeciale, AntonioHenderson, BillSpingler, BernhardHenderson, WilliamSpjuth, OlaHennig, AndreasSpooner, Robert AnthonyHenriques, CarlosSprio, Andrea ElioHepnarova, VendulaSreedasyam, AvinashHéquet, ValérieSreerama, SubramanyaHeredia-Guerrero, José AlejandroSri Vedavyasa Sri, RadhakrishnanHernández, FranciscaSridhar, JayalakshmiHernández-Alcoceba, RubénSrinivas, KeerthiHernández-Barajas, JoséSrivastava, AnupHernández-Castellano, Lorenzo E.Srivatsan, Eri S.Herndon, James W.Srivenugopal, KalkunteHerranz, Maria AngelesSroka, ZbigniewHerrera, RaquelStaal, JensHerrero, LauraStadler, MarcHerres-Pawlis, SonjaStagos, DimitriosHerrmann, FabianStaneva, GalyaHeys, JeffreyStaniszewska, MonikaHickey, Michael J.Stano, PasqualeHidalgo Herrador, José MiguelStanovnik, BrankoHideg, KálmánStanton, Daniel E.Higuchi, TsunehikoStara, IrenaHikawa, HidemasaStarek, MałgorzataHillwig, MatthewStarostik, PetrHingorani, DinaStavrianidi, AndreyHiorth, MarianneStedtfeld, RobertHirai, TomoyasuStefano, CaldaralliHirakawa, HidehikoStefanova, Natalia A.Hirakawa, KazutakaStefanovic, DarkoHirano, KojiSteinritz, DirkHirano, MasafumiStelzer, FranzHirano, TomoyaStenstrøm, YngveHirao, HajimeStephan, HolgerHirasawa, NoriyasuStephen, Michael RajeshHirst, Jonathan D.Štěpnička, PetrHisaki, IchiroSteunou, NathalieHo, Chi-TangSteup, MartinHo, Eric S.Stevanato, PiergiorgioHo, Wing ShingStevenson, StevenHocek, MichalStine, Keith J.Hochrainer, KarinStobiecka, MagdalenaHodges, RobertStobiecki, MaciejHodgkinson, JamesStock, ChristianHoffman, Angela M.Stockmann, ChristianHofmann, JohannStoikov, Ivan IvanovichHofmann, UteStojakowska, AnnaHogarth, GraemeStojan, JureHohsaka, TakahiroStrehlitz, BeateHojjat-Farsangi, MohammadStrekowski, LucjanHolalu, SrinidhiStride, John ArronHolder, Alvin A.Stromberg, RogerHollenstein, MarcelStrube, JochenHolopainen, JarmoStruga, MartaHolý, RichardŠtrukil, VjekoslavHolynska, MalgorzataStuart, JeffHoma, JoannaStunes, Astrid KamillaHomaeigohar, ShahinSturm, SonjaHoncharenko, MalgorzataSturtevant, JoyHong, HaoSu, Jui-HsinHong, Jiann-RueySu, Ming-DerHong, JinkeeSu, Neil QiangHong, YonggeunSu, PeifengHong, YuningSu, ZhiqiangHöpfl, HerbertSudarikov, Denis V.Horax, RonnySuga, SeijiHori, YuichiroSugahara, TakuyaHorie, MasanoriSugai, TakeshiHorino, YoshikazuSuganuma, SatoshiHorn Jr, AdolfoSugawara, AkihiroHorn, AnselmSugimoto, SachikoHorrigan, Louise A.Sugimura, HaruhikoHorvath, David P.Sugiura, MasaharuHorvath, DragosSuh, Hyung JooHosohata, KeikoSuh, Jun GyoHosokawa, SeijiroSuib, StevenHosoya, TakamitsuSuliburska, JoannaHosoya, TakashiSuliburska, Joanna MariaHossain, MohammadSuliman, SalwaHou, HarveySullivan, DevinHou, JingweiSumitani, Jun-ichiHoujou, HirohikoSun, DiHoung, Jer-YiingSun, GuohuiHour, Mann-JenSun, JingjingHowes, Laurence GuySun, LitaoHoyos, PilarSUN, MengHrabal, RichardSun, MingHricovÍni, MilošSun, PeilongHrnčič, Maša KnezSun, ShiHsiao, Sheng-HueiSun, WujinHsieh, Hsi-LungSun, YuanHsieh, Jung-FengSunatsuki, YukinariHsin, Kun-YiSung, Ping-JyunHsin, Ling-weiSung, Wen-ChiehHsu, Hsiu-FuSunna, AnwarHsuan, J.Supuran, Claudiu T.Hu, Ai-xiŠurinová, MáriaHu, Ching-YaoSurup, FrankHu, FangzhongSutter, ElianeHu, JinlianSutton, TroyHu, ShanghsiuSuturina, ElizavetaHUA, RuimaoSuzuka, ToshimasaHuang, ChangjiangSuzuki, Jon Y.Huang, Chi-ChangSuzuki, TakafumiHuang, Chiung-ChengSvendsen, John S.Huang, Chun-YungSvetashev, VasilyHuang, FeiheSvoronos, ParisHuang, Guan-JhongSwevers, LucHuang, Huang-ChiaoSwiatek, LukaszHuang, Hui-ChiŚwiątek, ŁukaszHuang, Jiann-JyhŚwiderska-Dąbrowska, RenataHuang, LeiSwiezewska, EwaHuang, Lin-FangSychrovsky, VladimirHuang, Tzou-ChiSydnes, Magne O.Huang, Wei-JanSyed, ViqarHuang, Wen-HsinSykes, CÉCILEHuang, WenlinSykes, MelissaHuang, Xue-shiSylwester, SlusarczykHUBER, ImreSynytsya, AndriyHübner, FlorianSzacon, ElzbietaHuefner, AntjeSzakonyi, ZsoltHuettmann, FalkSzczolko, WojciechHuh, Jin HoeSzekely, GyorgyHuh, Yun SukSzewczyk, KatarzynaHui, Kam M.Szkudelski, TomaszHuijbers, PatriciaSzopa, AgnieszkaHulme, ChristopherSzostak, MichalHung, Jan-jongSztanke, MałgorzataHung, Kuo-HsiangSzukalski, AdamHunter, LukeSzumny, AntoniHunyadi, AttilaTabata, JunHur, Jae-SeounTabata, YasuhikoHur, Sun-JinTabrez, ShamsHusnjak, KoraljkaTabrizi, Mojgan AghazadehHussein, Ahmed A.Tachibana, ShinjiroHussein, WaleedTaddei, MaurizioHuwiler, AndreaTadić, VanjaHwang, Bang YeonTagliatesta, PietroHwang, In KooTaherzadeh, Mohammad JavadHwu, Jih-RuTaira, JunseiHyeon, ChangbongTajmir-Riahi, H.A.Iacomini, MarcelloTak, Jun-hyungIannazzo, DanielaTakafumi, ItohIanora, AdriannaTakahashi, DaisukeIatrou, HermisTakahashi, KoretaroIbrahim, SalamTakahashi, ShoujiIchikawa, HiroshiTakanami, ToshikatsuIciek, MałgorzataTakano, KatsuraIdrissi, AbdenacerTakasu, KiyoseiIglesias, Luis E.Takata, GoroIglesias, Maria Del RosarioTakayama, FusakoIglesias-Fernández, RaquelTakayanagi, ToshiyukiIguchi, TaisenTakeda, SoichiIkai, TomoyukiTakenaga, MitsukoIkebukuro, KazunoriTakeoka, ShinjiIkehara, KenjiTakeshita, FumitakaIliopoulos, KonstantinosTakeuchi, DaisukeIlluminati, GiulioTakizawa, ShinobuImai, HiroyukiTalele, Tanaji TImaz, InharTamaoki, MasanoriImhof, PetraTamašauskaitė-Tamašiūnaitė, L.Imperatore, ConcettaTamura, MasazumiIndiveri, CesareTan, Le-HeInés Isla, MaríaTanaka, HiroyukiInguimbert, NicolasTanaka, HitoshiIn-Jung, KimTanaka, KazuoInman, SharonTanaka, MitsuruInokuchi, TsutomuTanaka, ShigenoriInuzuka, ToshiyasuTanaka, ShinjiIorio, EgidioTanaka, TakayukiIorizzi, MariaTanaka, TakujiIppoliti, ThomasTančinová, DanaIraqi, AhmedTang, BenjaminIsani, GloriaTaniguchi, NobukazuIshimizu, TakeshiTaniguchi, TohruIskandar, Michele M.Tanner, Julian A.Islam, M. NurulTantillo, DeanIsmaili, LhassaneTapia, Alejandro A.Isman, Murray B.Tarantino, GiovanniIsshiki, YoshiakiTarasova, Nadya I.Ita, KevinTaratula, OlenaIto, Jun-ichiTarr, D EllenIto, ShingoTarr, Matthew A.Ito, TakeruTarres, QuimIto, TakuyaTasca, CarlaIto, YoichiroTashima, KimihitoItoh, ToshiyukiTashima, ToshihikoItou, JunjiTashiro, EtsuIvancic Santek, MirelaTassieri, ManlioIvankin, AndreyTaura, FutoshiIvanov, Alexander VTava, AldoIvanov, AndreyTavares, AnthonyIvanov, MilanTavío, María Del MarIvanov, SergeyTawfik, Sherif AbdulkaderIvanova-Petropulos, VioletaTayebati, Seyed KhosrowIvashchenko, OlenaTaylor, CarolineIwamori, MasaoTaylor, James E.Iwaoka, MichioTchorbanov, AndreyIwasaki, TakashiTe Velthuis, AartjanIwata, AtsushiTeale, AndrewIZAWA, HironoriTecilla, PaoloJackson, William F.Teeri, TeemuJacobsen, Elisabeth EgholmTeixeira, RóbsonJadeja, RavirajsinhTellez, Trinidad RuízJadwicienczak, WojciechTellone, EsterJaenicke, StephanTelysheva, GalinaJAFRA, SylwiaTempesta, MariaJäger, MartinTeng, Yen-niJagerovic, NadineTerent’ev, Alexander O.Jagiello, KarolinaTeresa Del, Castillo-SantaellaJahandideh, SamadTerzaghi, WilliamJaiswal, Amit K.Teschke, RolfJakob, PeterTeufel, RobinJamart-Grégoire, BrigitteTheile, DirkJames, MargaretThérèse Charles, MarieJandrić, ZoraThiel, GeraldJang, Hye-YoungThiem, JoachimJános, HalászThiermann, HorstJanowski, MiroslawThierry, LangerJansen, RolfThiéry, ValérieJantas, D.Thijs, SofieJanuchowski, RadosławThiriet, MarcJaradat, NidalThomas, Eric J.Jara-Palacios, M. JoséThomas, JimJasiecki, JacekThomas, RobertJasiński, RadomirThomas, T. JohnJaumot, JoaquimThorek, Daniel L. J.Jazirehi, AliThünemann, AndreasJean, LudovicThuo, MartinJeandet, PhilippeThüs, HolgerJedlovszky, PalTidwell, CynthiaJędrzejczyk, Roman J.Tiera, MarcioJee, JunGooTietjen, IanJefferies, L.K.Tigini, ValeriaJelen, HenrykTihkonov, VladimirJenett-Siems, KristinaTimoshenko, Alexander V.Jensen, Henrik H.Ting, Richard C. H.Jenssen, HåvardTischler, DirkJeon, JunhaTitorenko, Vladimir I.Jeon, Sung HoTiwari, Rakesh KumarJeong, Lak ShinTkac, JanJeremy, TEOH Yuen ChunTo, Kenneth K WJerković, IgorToden, ShusukeJeschke, MarcTodorovic, TamaraJesionowski, TeofilToida, ToshihikoJeske, WalterTokitoh, NorihiroJeszka-Skowron, MagdalenaTokunaga, YujiJha, AmitabhTokuyama, ShogoJha, Kshitij C.Tollerup, KristenJi, DebinTolosa, EzequielJia, Yi-XiaTolstoy, Peter M.Jiahui, ShaoToma, LucioJiang, KeTomalia, DonaldJiang, LiuweiTomašič, TihomirJiang, XuchuanTomaz, Cândida TeixeiraJiang, YiTombelli, SaraJiang, ZuoquanTomczyk, MichałJiménez, Jordi JuárezTomi, FélixJiménez-Aparicio, ReytesTomitaka, AsahiJimenez-Arellanes, AdelinaTomoda, HiroshiJiménez-Aspee, FelipeTomofuji, TakaakiJiménez-Ballesta, RaimundoTomohiko Fukuda, TomohikoJiménez-Barbero, JesúsTömösközi, SándorJimeno, CirilTomovska, RadmilaJin, JiefuTonelli, Alan EdwardJin, MingTong, ShengJirkovsky, EduardToohey, JohnJirovetz, LeopoldTopic, FilipJjemba, Patrick K.Torigoe, KanjiroJo, Young SukTorikai, KoheiJoão Pires, Da SilvaTormo, José RubénJoers, ValerieTorrens, FranciscoJohánek, ViktorTorrents, EduardJohnston, Martin R.Torres Lagares, DanielJokela, JouniTorres Vaamonde, Jose EnriqueJones, JeremyTorres, Carlos F.Jones, KathrynTosheva, LubomiraJones, MarjorieToshio, KoizumiJonnalagadda, Sreekantha BabuTosi, PaolaJoo, Hong-GuTossi, AlessandroJoos, JonasTovoli, FrancescoJordan, V. CraigToyofuku, MasanoriJordão, António ManuelTrabocchi, AndreaJørgensen, KåreTran, CuongJosé Hipólito, Isaza MartínezTrandafirescu, CristinaJosue, DelgadoTrant, JohnJoule, JohnTravlos, IliasJózefowski, SzczepanTretyakova, Elena V.Juang, Shin-HunTrevitt, AdamJuarranz, ÁngelesTricoire, LudovicJudson, Richard STrif, MonicaJulianna, OláhTrincavelli, Maria LetiziaJuliano, ClaudiaTrincone, AntonioJullian, NathalieTrindle, CarlJullian, ValérieTringali, CorradoJung, Chol-HeeTripathi, AshootoshJung, JaehanTripodi, GianlucaJung, KlausTripp, Carl P.Jung, Sang HunTrotta, FrancescoJung, SeunhoTrujillo-Rodríguez, María JoséJung, Young-SukTruong Phuoc, NghiaJunghwan, OhTrusheva, BoryanaJurikova, TundeTrusso Sfrazzetto, GiuseppeJurman, GiuseppeTrzeciak, A. M.Juskowiak, BernardTrzeciak, Anna M.Juszczak, LesławTsai, Shau-WeiJuvik, JohnTsai, Teh-huaK. Lauf, PeterTsai, YenlingKabala-Dzik, AgataTsai, YingChiehKabil, OmerTsakalof, AndreasKachamakova-Trojanowska, NeliTsaltas, DimitrisKachlicki, PiotrTsantili-Kakoulidou, AnnaKaczor, Agnieszka A.Tseng, Chih-HuaKadakowa, Prof. Jun-ichiTseng, Ling-MingKadela-Tomanek, MonikaTsipis, Athanassios C.Kaden, PeterTsopmo, ApollinaireKafarski, PawełTsotinis, AndrewKafle, LekhnathTsoungas, Petros G.Kagan, Isabelle A.Tsow, Francis (Tsing)Kalantar-Zadeh, KouroshTsubaki, KazunoriKalayda, AnyaTsugawa, HiroshiKalhapure, Rahul S.Tsuji, PetraKalhotka, LiborTsukuda, KazunoriKalikova, KvetaTuccinardi, TizianoKalinin, Vladimir I.Tulkens, PaulKalinowska Lis, UrszulaTullio, VivianKaliszan, RomanTullius Scotti, MarcusKalogirou, CharisTunaru, SorinKamagata, KiyotoTung, Chih-KuanKamdem, Jean PaulTung, Chun-WeiKamdem, PascalTurano, PaolaKan, Chi-waiTurco, AntonioKanakkanthara, ArunTurkowski, VolodymyrKananovich, Dzmitry G.Turley, Eva A.Kanda, YasuharuTurło, JadwigaKang, Bum-yongTurrini, EleonoraKang, ChanKyuTuttolomondo, AntoninoKang, ChulhunTylewicz, UrszulaKang, CongbaoTylkowski, BartoszKang, Hee EunTyner, Jeffrey W.Kang, Jihee LeeTyzack, JonathanKang, Jong SeongTzakos, Andreas G.Kang, JonghoonTzounis, LazarosKang, SinyoungTzur, EyalKang, WenyiUbukata, TakashiKang, Young-HeeUccello Barretta, GloriaKankanala, JayakanthUccello-Barretta, GloriaKanna, MachiUchi, HiroshiKannan, MeganathanUddin, ShaikhKantminienė, KristinaUekusa, HidehiroKanzaki, HiroyukiUemura, KazuhiroKao, Chai-LinUeng, Yune-FangKapalavavi, BrahmamUfnal, MarcinKarabagias, IoannisUggerud, EinarKarafiloglou, PadeleimonUllah, AmanKaragoz, KubraUlrich, DetlefKarakasidis, T.E.Umeyama, AkemiKarakhanov, EduardUno, TomohideKaramanoli, KaterinaUpare, Pravin P.Karanis, PanagiotisUrbano, A. M.Karchesy, JoeUribe-Romo, Fernando J.Kar-Chun, TanUrieta, JoséKarim, Mohammad RezaulUrra, FelixKarlic, HeidrunUsai, MariannaKårlund, AnnaUsami, YoshihideKarmakar, ParthaUsuki, ToyonobuKarnaouri, AnthiUto, YoshikazuKarpiński, Tomasz M.Uzio, DenisKarpoormath, RajshekharVáclav, BrázdaKarran, PeterVago, RiccardoKarsili, TolgaVähä-Koskela, MarkusKaruso, PeterVaidya, AmitaKashfi, KhosrowValable, SamuelKashiwagi, ShinichiroVale, NunoKashman, YoelValencia, GregorioKaškonienė, VilmaValentão, PatríciaKasoju, NareshValentová, KateřinaKataev, EvgenyValentovic, MonicaKatagiri, HiroshiValenzuela, RodrigoKataoka, H.Valero, ManuelKatayama, ShigeruValkó, Klára L.Katiyar, Santosh K.Valletta, AlessioKato, MasayaVallon, VolkerKatona, GabrielVan Aerschot, ArthurKatsantonis, DimitriosVan Der Auweraer, M.Katsila, TheodoraVan Der Hooft, JustinKaufmann, DorotheaVan Der Kuyl, AntoinetteKauppinen, AnuVan Der Straeten, DominiqueKaur, SumanjeetVan Griensven, LeoKaushiK, AjeetVan Herwijnen, HendrikKawabata, KyuichiVan Kampen, Jackalina M.Kawada, ManabuVan Lanen, StevenKawaguchi, Shin-ichiVan Scherpenzeel, MoniqueKawai, YoshichikaVanden Eynde, Jean JacquesKawakami, SusumuVandenbussche, FilipKawakami, YukiVandooren, JenniferKawakita, HidetakaVanelle, PatriceKawamura, AkiraVanEpps, J. ScottKawamura, IzuruVangala, VenuKawano, KouichiroVannier, Rose-NoëlleKawao, NaoyukiVannini, AndreaKawase, MasamiVanoni, Maria AntoniettaKawato, YujiVarela, JoaoKawatsura, MotoiVarela-López, AlfonsoKayano, Shin-ichiVarela-Ramirez, ArmandoKeasar, ChenVarga, ElisabethKedage, VivekanandaVarga, ZoltanKeglevich, GyörgyVargas-Baca, IgnacioKeiser, MarkusVargiu, AttilioKellogg, GlenVárnai, AnikóKemp, BelindaVarvounis, GeorgeKerch, GarryVasaikar, SuhasKerr, William L.Vasicek, OndrejKerrihard, Adrian L.Vasile, CorneliaKerwin, Sean M.Vasile, FrancescaKessel, DavidVasilev Pavlov, DanailKeszler, AgnesVasilyev, AleksanderKevadiya, BhaveahVassaux, GeorgesKhadka, ManojVasto, SonyaKhalil, AbdelouahedVaz, JosianaKhan, IzharVázquez Tato, M. PilarKhan, MujeebVázquez-Flota, FelipeKhan, MushfiquddinVecino, XanelKhatri, KshitijVekariya, RakeshKhlestkina, ElenaVelazquez-Contreras, Carlos-arturoKhoo, YuehawVelez, ZéliaKhoobchandani, MenkaVelezheva, Valeriya S.Khotimchenko, Maxim Y.Velivelli, SivaKhrustalev, VictorVelliyagounder, KabilanKhurshid, ZohaibVemula, HarikaKicel, AgnieszkaVemulpad, Subramanyam RKieć-Kononowicz, KatarzynaVenditti, AlessandroKieliszek, MarekVenkataraman, ShrinivasKikelj, DanijelVennerstrom, JonathanKikuchi, HaruhisaVenter, RietieKikuchi, TakashiVenturi, FrancescaKikuzaki, HiroeVenus, JoachimKilian, KrzysztofVepsäläinen, JoukoKilleen, DanielVerardi, AlessandraKim, Chul YoungVerdolotti, LetiziaKim, Chun HoVerestiuc, LilianaKim, Chung SubVergadi, EleniKim, DaehwanVergara, DanieleKim, Dong SubVerma, Mohit S.Kim, DonginVerma, Navin KumarKim, DongukVerma, SannyKim, DoseokVermaas, Joshua V.Kim, HangunVernetti, Lawrence A.Kim, HojoongVerri Jr., Waldiceu A.Kim, HoonVerron, EliseKim, HyemeeVervoort, JacquesKim, HyesunVetvicka, VaclavKim, Hyeung-RakViapiana, AgnieszkaKim, HyoungsuVicent, CristianKim, Hyung SikVicente, ClaudiaKim, Hyun-PyoVieira Ribeiro, João RuiKim, HyunwooVieira, Margarida C.Kim, Ick YoungViggiano, AndreaKim, InKyeomVigier, KarineKim, JaheonVíglaský, ViktorKim, JooyounVila, CarlosKim, Jung-HoonVilela Sampaio, SuelyKim, JunheonVilela, AliceKim, Keun-SikVillacampa, Maria DoloresKim, Ki HyunVillain, SylvieKim, Kil-SooVillalobo, AntonioKim, Kwang PyoVillani, ClaudioKim, No SooVinardell, MariaKim, Sang HoonViñas, MiguelKim, Sang JaeVincent Y-H, ChengKim, Seok-JhinVincentini, OlimpiaKim, Soo-UnVirjamo, VirpiKim, Sung-HoonVirta, PasiKim, Sun-JuViscarra, JoseKim, Tae KonVisioli, FrancescoKim, TaewooVisioli, GiovannaKim, Won HoVistoli, GiulioKim, Woo-YoungVita, Giuseppe DiKim, YangVitali, FrancescaKim, YoungmeeVitalini, SaraKim, Yun-baeVladimirov, Vladimir I.Kim, YuriVlashi, ErinaKimber, Marc CVoglmayr, HermannKimura, HidetoVoliani, ValerioKimura, IkukoVon Hänisch, CarstenKimura, Ken-ichiVon Knethen, AndreasKimura, NobuyukiVon Wright, AtteKinder, DavidVorob’ev, Mikhail M.Kinoshita, TakayoshiVoulhoux, RomeKinthada, RamakumarVoutquenne, LaurenceKiparissides, CostasVriend, JerryKirsch, GilbertVucenik, IvanaKiselev, K.v.Vullo, DanielaKisiela, DagmaraVynios, DemitriosKiss, JánosWagner, AnikaKissin, Yury V.Wagner, BrianKita, YasuyukiWagner, Carl E.Kitagishi, HiroakiWähälä, KristiinaKitamura, MasanoriWajs-Bonikowska, AnnaKitano, YoshikazuWaksmundzka-Hajnos, MonikaKitazawa, TakafumiWalczak, Maciej A.Kiuru, PaulaWaldmeier, FelixKlajnert-Maculewicz, BarbaraWaldron, KevinKleczkowski, Leszek A.Walenga, JeanineKlegeris, AndisWalenga, Jeanine M.Kleier, Daniel A.Waleron, KrzysztofKlewicka, ElzbietaWalker, HeatherKlumpp, Douglas A.Wallace, DavidKluza, JéromeWallace, NicholasKnapp, SpencerWallin, ErikaKnippschild, UweWall-Medrano, AbrahamKnölker, Hans-JoachimWalton, JohnKnupp, GerdWaluk, JacekKnutsen, SveinWan, EdwinKo, Kam MingWan, JianboKo, Kyoung ChulWan, LeiKo, Shun-YaoWan, ShibiaoKobayashi, ShingoWan, XiaochunKobayashi, YoshiroWan, ZhongxiaoKobets, TetyanaWang, Bo-ChengKoch, EgonWang, ChaojieKocoglu, MehmetWang, Chien-LungKoes, David RyanWang, Ching-ChiungKoetz, JoachimWang, Chin-KunKofuji, KyokoWang, Chrong-ReenKogure, NoriyukiWang, CundeKokotkiewicz, AdamWang, Feng-ShengKokotos, Christoforos G.Wang, GuozhengKokubun, TetsuoWANG, HAOYUKolář, Michal H.Wang, JeffreyKolaric, BrankoWang, Jeh-jengKoller, MartinWang, Jian-WenKolodkin-Gal, IlanaWang, JianyingKomiyama, MakotoWang, JunjianKondo, Shin-ichiWang, JunjieKondoudakis, NikosWang, Kevin YuejuKong Thoo Lin, PaulWang, LeiKong, Chang-SukWang, LiliKong, In-sooWang, Meng-jiyKong, JinWang, NanKong, Ling BingWang, PengKönigs, Rene M.Wang, PengchengKonno, HiroyukiWang, PiwenKontogiorgis, ChristosWang, QunKopel, PavelWang, Robert Y. L.Kopka, KlausWang, RuibingKoppel, KadriWang, San-LangKopra, KariWang, ShengnianKornev, AlexandrWang, Sheng-ShihKorshun, VladimirWang, Sheng-YangKorzeniewska, EwaWang, Tzu-FanKos, GregorWang, WeiKoschella, AndreasWang, WentianKoshino, HiroyukiWang, XiaohongKosmala, MonikaWang, XiaoningKostakis, IoannisWang, XinwenKöster, FrankWANG, XizuKosters, AstridWang, XuanbinKostyuk, Vladimir A.Wang, YengtsengKotašková, JanaWang, YifeiKotsiantis, SotirisWang, YingKotsikorou, EvangeliaWang, YixiangKoufaki, MariaWang, YumengKoukounaras, AthanasiosWang, YunKouretas, DimitriosWang, Yun-MingKourkoutas, YiannisWang, ZhijunKoutelidakis, AntoniosWaszkielewicz, Anna M.Koutselas, IoannisWatanabe, MasatoshiKovacic, HervéWatanabe, RyuichiKovács, LajosWawer, IwonaKovalčík, AdriánaWebber, MarkKovalenko, AndriyWeber, FabianKowalak, StanisławWeber, JohnKowalewski, MarkusWefers, DanielKowalska, EwaWeger, StefanKowalska, IwonaWei, GangKowalska, KatarzynaWei, Guor-JienKowalska, TeresaWei, GuoweiKowalski, KonradWei, LeyiKowalski, RadosławWei, RenKoyani, ChintanWeidner, SteffenKoziak, KatarzynaWeiss, JuliaKozieł, EdmundWelch, Aaron Z.Koziel, JacekWelgamage Don, AakashKozliak, Evguenii I.Wellinger, Ralf-ErikKozlov, DenisWen, Amy M.Kozlova, Svetlana G.Weng, ChingfengKozłowska, MariolaWenthold, Paul GKozlowski, HenrykWentrup, CurtKrahl, ThoralfWertz, Philip W.Krämer, RolandWesolowski, MarekKrämer, StefanieWesolowski, RobertKregiel, DorotaWestcott, Stephen A.Kreiner, GrzegorzWestermann, BernhardKřen, VladimírWestwell, AndrewKries, HajoWestwell, Andrew D.Krol, EwelinaWhelan, Rebecca J.Kruger, JacobWhite, RobertKruk, Mikalai M.White, Robert L.Krumeich, FrankWianowska, DorotaKruppa, KlaudiaWiczk, WiesławKu, SeockmoWiczkowski, WieslawKuan, Yu-HsiangWidener, ChristianKubatka, PeterWie, Myung-BokKubička, DavidWieckowska, AgnieszkaKubicova, LenkaWieckowska, AnnaKubina, RobertWiedman, GregoryKubo, TakanoriWiese, SebastianKucharska, Alicja Z.Wilhelm, ReneKucuk, IsrafilWilhelm, StefanKuerten, StefanieWilkens, MirjaKuhnert, NikolaiWilkes, EricKujawska, MałgorzataWilkinson, BrendanKujawski, JacekWilkinson, J. M.Kujawski, WojciechWilkinson, John C.Kukula-Koch, WirginiaWilliams, Andrew R.Kuligowski, MaciejWilliams, David L.Kulma, AnnaWilliams, Noelle S.Kulma, MagdalenaWilliams, René M.Kumar, AshutoshWillner, ItamarKumar, ManuWilson, JulieKumar, NarendraWilson, Justin J.Kumar, PawanWin, Khin YinKumar, SajeeshWinckler, ThomasKumar, SushilWinkler, JohannesKumar, UjendraWińska, KatarzynaKundu, AishwaryaWiszniewska, AlinaKunjachan, SijumonWitczak, CarolKunnimalaiyaan, MuthusamyWitczak, ZbigniewKunsagi-Mate, SandorWitzgall, PeterKunz, WolframWlodawer, AlexanderKunze, GotthardWnuk, Stanislaw F.Künzler, MarkusWohleb, Eric S.Kuo, Chung-FengWojaczyńska, ElżbietaKuo, Ping-ChungWojcieszak, RobertKupihár, ZoltánWojcieszyńska, DanutaKurdowska, Anna K.Wojdyło, AnetaKuroboshi, ManabuWójtowicz, AgnieszkaKuroda, MinpeiWojtyla, ŁukaszKusano, ShuheiWölfle, UteKusch, PeterWolf-Rainer, AbrahamKuśmierek, KrzysztofWolfram, JoyKuwabara, MasanariWondim, Dessie SalilewKuwahara, MasayasuWong, Chung F.Kuźnik, NikodemWong, Ka-ChunKvasnica, MiroslavWong, Ka-LeungKwak, Jong HwanWong, Wallace W. H.Kwak, SeonyeongWoo, ChristinaKwakowsky, AndreaWoodward, CliffordKwan, Hiu-yeeWoodward, Robert T.Kwoh, Chee KeongWorek, FranzKwok, Ngai MingWoźniak, Łukaszkwok-wing, chauWoźniak-Budych, MartaKyzas, George Z.Woznicki, Tomasz L.La Motta, ConcettinaWu, BinLa Penna , GiovanniWu, Chi-ReiLaatsch, HartmutWu, DaochengLacaille-Dubois, Marie-alethWu, Ho-ShingLachman, JaromirWu, JianyongLachowicz, Joanna IzabelaWu, Jin-YiLachowicz, SabinaWu, Judy I.Lachter, Elizabeth R.Wu, Kevin C.-W.Ladero, MiguelWu, Kuen-phonLadurner, AngelaWu, Li-ChenLaffleur, FlaviaWu, Ming-JiuanLaforenza, UmbertoWu, NanLaghi, LucaWu, Sheng-ChiLaguna, MarianoWu, Shu-JuLahlali, RachidWu, Shu-PaoLai, QinghuaWu, Tian-ShungLaitinen, RiikkaWu, Tzi-YiLakowski, TedWu, Tzong-YuanLamba, DorianoWu, WeiLaMontagne, MichaelWu, Wen-LuanLamprou, DimitriosWu, WenruLan, TianWu, YingLan, WenjianWu, ZhanhongLan, YuWu, ZhenghongLancelin, Jean-MarcWu, ZhiShengLangdon, Simon P.Wujec, MonikaLange, HeikoWullaert, AndyLangford, Cooper H.Wydra, KerstinLaplante, MathieuWyszko, ElizaLarena, InmaculadaXavier, Cristina Pinto RibeiroLaRock, Christopher N.Xavier, Nuno ManuelLaronze-Cochard, MarieXi, WeixianLarraneta Landa, EnekoXia, AndongLarsen, Ole HalfdanXiang, ChunhuiLarson, Nicholas B.Xie, GuoxiangLaRue, JerryXie, HaoranLash, TimothyXie, JianpingLatosińska, Jolanta NataliaXie, RanLaubach, Victor E.Xie, Zhong-RuLaube, MarkusXin, DongyueLauder, BobXing, ChengguoLaudone, GiulianoXing, XiaohuiLaunay, Jean MarieXiong, AishengLauritano, DorinaXiong, HaifengLauschke, VolkerXiong, ZhaohuiLavandera, IvánXodo, LuigiLavarda, Francisco CarlosXu, DazhongLavelli, VeraXu, XiaoliangLaverty, GarryXu, XudongLavorgna, MarinoXu, XuemingLavrik, Inna N.Xu, YanLAW, Ka HoXu, ZhenghuLayek, BuddhadevXuan, Tran DangLazzara, GiuseppeYakura, TakayukiLazzarato, LorettaYamada, KojiLe Grognec, ErwanYamada, ShuheiLe Hégarat, LudovicYamada, SohsukeLe Pogam, PierreYamagishi, TakehiroLe Roes-Hill, MarilizeYamaguchi, AritomoLe, DuyYamaguchi, FumioLeahy, JamesYamaguchi, KanjiLeal, Ana MendesYamaguchi, SeijiLebaron, RichardYamaki, KohjiLebeau, LucYamamoto, YasunoriLebedev, Albert T.Yamana, KazushigeLeboffe, LorisYamato, TakehikoLecca, PaolaYamauchi, SatoshiLechtenberg, MatthiasYan, DongqingLee, Bong HoYan, HaiLee, Boo-YongYan, Hongbin (Tony)Lee, Bun YeoulYan, KaiLee, Byung-HeonYan, XiaohuiLee, Chang HoonYan, XuehuaLee, Che-HsinYanagita, Ryo C.Lee, ChiYancheva Pantaleeva, DenitsaLee, Chia-HungYang, Chin-YingLee, Ching-KuoYang, Dae HyeokLee, Chong-KilYang, Ding-YahLee, DonghanYang, FafuLee, Geun-ShikYang, GuangdongLee, Hae-HyoungYang, HangshengLee, HeesoonYang, HongshunLee, Hye SukYang, HuaiyuLee, Hyun SooYang, JaemoonLee, Jae HwanYang, JunLee, Jae WookYang, LijiangLee, JaehwiYang, Pei-MingLee, JeongmiYang, QiyuanLee, Jin-KooYang, TaoLee, Jiun-hawYang, Tian J.Lee, JooyoungYang, Wei-hsiungLee, Jun HeeYang, Xiu weiLee, Jun SikYang, YongkangLee, JunguYang, Yu-ChiaoLee, Keun-HyeungYang, Yu-liangLee, Kwang HoYang, ZhenLee, Mei-HsienYang, ZhijunLee, Mi KyeongYao, JianxiuLee, Min WonYao, WeirongLee, Sang GilYao, YanhuaLee, Sang YeolYao, ZhiliLee, Sang-HanYao, Zhong-KeLee, Seung HeeYaremenko, Ivan A.Lee, Seung-HongYaseen, AshrafLee, Shyi-LongYasuike, MotoshigeLee, SunwooYasunami, MichioLee, Tae SooYates, Edwin A.Lee, Vladimir Ya.Yates, Matthew Z.Lee, Yong RokYe, HaobinLee, Yun-SangYe, LongLee, ZhenghongYe, ZhouLefebvre, FredericYeh, Jan-yingLegault, JeanYeh, Jui-MingLegentil, LaurentYen, Chia-HungLehnherr, DanYen, Ming-ShienLei, Xin GenYentekakis, Ioannis V.Leigh, William J.YERABOLU, JAYASUDHAN REDDYLeiherer, AndreasYi, MyunggiLein, MatthiasYi, Young-SuLeite, Edda LisboaYiannakopoulou, EugeniaLekka, MalgorzataYin, GuoweiLemaire, Martin T.Yin, JianLEMEUNE, AllaYin, JunLemoine, PierricYin, RuoheLen, ChristopheYin, ShengLenardao, EderYin, ShiLenardão, Eder J.Ying, Gui-shuangLenaz, GiorgioYogendra, KalenahalliLeng, RogerYohei, ShirakamiLentini, GiovanniYokoi, TsuyoshiLeon, CarlosYokomatsu, TsutomuLeon, JosefaYokosuka, AkihitoLeong, David TaiYokota, ShinsoLeoni, AlbertoYokoya, MasashiLeonidas, Demetres D.Yokoyama, KenichiLeon-Reyes, AntonioYonemochi, EtsuoLeón-Rivera, IsmaelYoneshiro, TakeshiLephart, Edwin D.Yoo, Eun-SookLepoittevin, BénédicteYoon, In-SooLeporatti, StefanoYoon, Kee DongLeroux, FrédéricYoon, Moon-YoungLeroy, BaptisteYoon, Tae-HoonLesch, AndreasYoshida, JunLesner, AdamYoshida, KumiLesyk, Roman B.Yoshii, HidefumiLeung, Chung-HangYoshiji, OhtaLeung, IvanhoeYoshimoto, MakotoLeung, PollyYoshimura, MorioLeung, Ricky Yuet-KinYoshinari, TomoyaLevaj, BrankaYoshitake, HideakiLeveneur, SébastienYou, MingxuLevi-Polyachenko, NicoleYoung, Douglas D.Lew, D. BettyYoza, Brandon A.Lewinska, AnnaYperman, JanLewis, RandolphYu, ChenglongLewkowski, JaroslawYu, Chia-LiLeys, DavidYu, FabiaoLherbet, ChristianYu, HuaLhiaubet, Virginie LyriaYu, MeihuaLi, BoYu, RichardLi, CalvinYu, WenquanLi, ChunshunYu, Yan PingLi, DegangYu, YangLi, DongyeYUAN, TAOLi, FengYuan, YaxiaLi, Hou-JinYuan, YonganLi, Hua-BinYuan, Ze-ChunLi, JieYuan, ZhihongLi, Jie JackYuba, EijiLi, JinghaoYue, YuanzhengLi, Jin-HengYumoto, HiromichiLi, Kuo-BinYu-Tse, WuLi, LongyuZabetakis, IoannisLi, Mei-LingZabielska-Koczywąs, KatarzynaLi, NianguangZaccaro, LauraLi, PingZacharias, MartinLi, Shao-GangZacharov, SergejLi, TianhuZaferanloo, BitaLi, Xiang-GuoŽagar, EmaLi, XiaohuaZagotto, GiuseppeLi, YanZaharia, ValentinLi, YangZahn, Jeffrey D.Li, YifanZaid, HilalLi, YinZakrzewski, JanuszLi, YingZakrzewski, JerzyLi, YiwenZaleska-Medynska, AdrianaLi, ZhaohuiZamaratskaia, GaliaLi, ZhonghuZamò, AlbertoLi, ZibiaoZamyatina, AllaLiagre, BertrandZamyatnin, Andrey A.Liang, DongZanetti, MichelaLiang, GuodongZang, LiqingLiang, Steven H.Zannas, Anthony S.Liang, YongZappia, GiovanniLiang, YuerongZaprutko, LucjuszLianos, PanagiotisZarejousheghani, MashaalahLiao, Jiahn-HaurZarra, IgnacioLiao, Jyh-feiZarrelli, ArmandoLiao, Pei-ChunZarrouk, OlfaLichtman, MartinZarubaev, VladimirLidon, FernandoZavialov, AntonLikodimos, VlassiosZbigniew, SrokaLiljeblad, ArtoZdarta, JakubLim, Ji-HongZeidan, YoussefLim, Sung-wooZeilinger, CarstenLim, Yong-BeomZeitler, AxelLim, YoonghoZeković, ZoranLima-Neto, BeneditoZeleny, ReinhardLin, ChenZelko, RomanaLin, Chi-ChenZeng, HuaqiangLin, GialihZeng, HulieLin, Hai-shuZeng, XiangxiangLin, HaoŻesławska, EwaLin, HuiZgórka, GrażynaLin, Kuo-ShyanZhai, Hong LinLin, Liang-TzungZhang, BoLin, Li-GenZhang, CaiguoLin, Long-LiuZhang, DaweiLin, Mei-HsiangZhang, DeqiangLin, Pei-HuiZhang, FanLIN, Sze Ki CarolZhang, GeLin, Yang-weiZhang, Hong-jieLinard, ChristineZhang, HuaweiLindel, ThomasZhang, JianLindemann, PeterZhang, JianyingLindèn, MikaZhang, JieLinder, StigZhang, JingjingLindoy, Leonard F.Zhang, Jun-LongLindsey, JonathanZhang, JunzengLindsey, Jonathan S.Zhang, KaiLinert, WolfgangZhang, LiangrenLing, ChenZhang, LibingLinhardt, RobertZhang, LongheLinton, Brian R.Zhang, Ming-RongLiou, Ying-mingZhang, QianqianLipke, Peter N.Zhang, QingzhuLipok, JacekZhang, RuiyongLippens, GuyZhang, RunLiptrott, NeillZhang, ShaozhongLitinas, KonstantinosZhang, ShuLitvić, MladenZhang, WeiminLitwinienko, GrzegorzZhang, WenhengLiu, BifengZhang, WujieLiu, BinZhang, YanminLiu, Cheuk LunZhang, YingKaiLiu, ChuanjunZhang, YingyueLiu, DelongZhang, YixiangLiu, FeiZhang, YonghongLiu, HaipengZhang, YuhangLiu, JinyunZhang, YunkaiLiu, JunZhang, Z.Liu, Jun-JenZhang, ZehuiLiu, LeiZhang, ZhenbinLiu, MeifengZhang, ZhenhuaLiu, Shing-HwaZhao, BinLiu, Shiuh-TzungZhao, HuabinLiu, ShuyingZhao, MingLiu, WeiZhao, Qin-ShiLiu, XiaoZhao, WeifengLiu, XiaojingZhao, WENXIULiu, XiaoxiZhao, YuxiaLiu, YangZhbannikov, Ilya Y.Liu, YonghongZheng, ShilongLiu, YongqiangZheng, XinyuanLiu, YongqingZheng, YongtangLiu, YuanhongZhong, TuhuaLiu, YujunZhou, Guang-BiaoLiu, YunlingZhou, Hai-BingLiu, ZhangZhou, JiaLiu, ZhiqiuZhou, JiajingLiu, ZhixiaZhou, LeiLiva Rakotondraibe, HarinantenainaZhou, QingxiangLlewellyn, Lyndon E.Zhou, WenchaoLlorens, EugenioZhou, YifaLlorent-Martínez, Eulogio J.Zhou, ZheLloyd-Williams, PaulZhu, ChengjianLo Presti, LeonardoZhukov, IgorLo Scalzo, RobertoZhukova, NataliaLo, Huang-MuZi, XiaolinLo, SzechengZia-Ul-Haq, MuhammadLo, Yi-chenZięba, AndrzejLöbmann, KorbinianZiegler, JörgLøbner-Olesen, AndersZiegler-Borowska, MartaLocatelli, MarcelloZielińska, SylwiaLockridge, OksanaZielińska-Pisklak, Monika A.Lodyga-Chruscinska, ElzbietaZimmerman, Paul M.Lognay, GeorgesZimmerman, StevenLohézic-Le Dévéhat, FrançoiseZingue, StéphaneLoiseau, Philippe M.Zitko, JanLoke, Wai MunZłotek, UrszulaLong , Timothy E.Żołnowska, BeataLong, BrianZonta, CristianoLongo, PasqualeZorc, BrankaLonnberg, HarriZordoky, BeshayLopes, Carla M.Zou, QuanLopes, Lucia Maria XavierZoumpanioti, MariaLopez Rodilla, Jesus M.Zoupa, MariaLópez, ConcepciónZucca, PaoloLopez, CristobalZüllig, RichardLopez, Dolores E.Zuo, JianLópez, José Antonio PalenzuelaZuo, LiLoppi, StefanoZupko, IstvanLoquet, AntoineZuppinger, ChristianLorberboum-Galski, HayaZutz, ChristophLorenzi, VanninaZwanenburg, BinneLorenzo Tendero, CándidaZygmunt, MałgorzataLotan, NoahZykwinska, AgataLou, SongŻyżelewicz, DorotaLouis, Sandrine

